# New records and detailed distribution and abundance of selected arthropod species collected between 1999 and 2011 in Azorean native forests

**DOI:** 10.3897/BDJ.4.e10948

**Published:** 2016-12-22

**Authors:** Paulo A.V. Borges, Clara Gaspar, Luís Carlos Fonseca Crespo, François Rigal, Pedro Cardoso, Fernando Pereira, Carla Rego, Isabel R. Amorim, Catarina Melo, Carlos Aguiar, Genage André, Enésima P. Mendonça, Sérvio Ribeiro, Joaquín Hortal, Ana M.C. Santos, Luís Barcelos, Henrik Enghoff, Volker Mahnert, Margarida T. Pita, Jordi Ribes, Arturo Baz, António B. Sousa, Virgílio Vieira, Jörg Wunderlich, Aristeidis Parmakelis, Robert J. Whittaker, José Alberto Quartau, Artur R.M. Serrano, Kostas A. Triantis

**Affiliations:** 1 cE3c – Centre for Ecology, Evolution and Environmental Changes / Azorean Biodiversity Group and Universidade dos Açores - Departamento de Ciências e Engenharia do Ambiente, Rua Capitão João d’Ávila, São Pedro, 9700-042 Angra do Heroísmo, Terceira, Azores, Portugal; 2 Departament de Biologia Animal and Institut de Recerca de la Biodiversitat (IRBio), Universitat de Barcelona, Avinguda Diagonal 643, 08071, Barcelona, Spain; 3 Environment and Microbiology Team, IPREM UMRCNRS-UPPA 5254, IBEAS BP1155, Université de Pau et des Pays de l’Adour, 64013 Pau Cedex, France; 4 Finnish Museum of Natural History, University of Helsinki, Pohjoinen Rautatiekatu 13, P.O.Box 17, 00014, Helsinki, Finland; 5 cE3c, Centre for Ecology, Evolution and Environmental Changes & Faculty of Sciences, University of Lisbon, 1749-016, Lisbon, Portugal; 6 Laboratório de Ecologia Evolutiva de Herbívoros de Dossel, DEBIO, Instituto de Ciências Exatas e Biológicas, Universidade Federal de Ouro Preto, Campus Morro do Cruzeiro, 35400-000, Ouro Preto, MG, Brazil; 7 Departamento de Biogeografía y Cambio Global, Museo Nacional de Ciencias Naturales (CSIC), C⁄Joseé Gutiérrez Abascal 2, 28006, Madrid, Spain; 8 Natural History Museum of Denmark, University of Copenhagen, Universitetsparken 15, DK-2100, Copenhagen OE, Denmark; 9 Museum d´Histoire Naturelle, Case Postale 6434, 1211, Geneva, Swaziland; 10 Centro de Estudos da Macaronésia (CEM), Universidade da Madeira, Campus Universitário da Penteada - Bloco C - Piso 1, 9000-399 Funchal, Madeira, Portugal; 11 Valencia 123-125, ent., 3a, E-08011, Barcelona, Spain; 12 Dep. de Ciencias de la Vida. Universidad de Alcalá, 28871 Alcalá de Henares, Madrid, Spain; 13 SPEN – Sociedade Portuguesa de Entomologia, Apartado 8221, P-1803-001, Lisboa, Portugal; 14 Departamento de Biologia, Universidade dos Açores, Apartado 1422, 9501-301, Ponta Delgada, S. Miguel, Azores, Portugal; 15 Hindenburgstr. 94, D-75334, Straubenhardt, Germany; 16 Department of Ecology and Taxonomy, Faculty of Biology, National and Kapodistrian University of Athens, Athens, GR-15784, Greece; 17 Biodiversity Research Group, Oxford University, Centre for the Environment, South Parks Road, Oxford, OX1 3QY, United Kingdom

**Keywords:** Azores; terrestrial arthropods; BALA project; laurissilva forest; Linnean, Wallacean and Prestonian shortfalls.

## Abstract

**Background:**

In this contribution we present detailed distribution and abundance data for arthropod species identified during the BALA – ***B****iodiversity of **A**rthropods from the **L**aurisilva of the **A**zores* (1999-2004) and BALA2 projects (2010-2011) from 18 native forest fragments in seven of the nine Azorean islands (all excluding Graciosa and Corvo islands, which have no native forest left).

**New information:**

Of the total 286 species identified, 81% were captured between 1999 and 2000, a period during which only 39% of all the samples were collected. On average, arthropod richness for each island increased by 10% during the time frame of these projects. The classes Arachnida, Chilopoda and Diplopoda represent the most remarkable cases of new island records, with more than 30% of the records being novelties. This study stresses the need to expand the approaches applied in these projects to other habitats in the Azores, and more importantly to other less surveyed taxonomic groups (e.g. Diptera and Hymenoptera). These steps are fundamental for getting a more accurate assessment of biodiversity in the archipelago.

## Introduction

In 1999 a group of researchers from the University of the Azores and the University of Lisbon started a long-term (1999-2004) standardized sampling program to inventory the arthropod biodiversity in native forest remnants of the Azores - the BALA I project – ***B****iodiversity of **A**rthropods from the **L**aurisilva of the **A**zores* (Borges et al. 2000, Borges et al. 2005a, Borges et al. 2011, Ribeiro et al. 2005, Gaspar et al. 2008). More recently, this project was extended by researchers from the Universities of the Azores, Athens and Oxford, by surveying part of the same native forest plots almost 10 years later - BALA II project (2010-2011).

Eight years of standardized survey of the native forest in seven of the nine Azorean islands resulted in a major improvement on the knowledge of the Azorean arthropod fauna, in particular concerning Araneae, Opiliones, Pseudoscorpionida, Diplopoda, Chilopoda and Insecta (excluding Collembola, Diptera and Hymenoptera). As a consequence, several new endemic taxa were described for the archipelago (e.g. Blas and Borges 1999, Ribes and Borges 2001, Platia and Borges 2002, Quartau and Borges 2003, Borges et al. 2004, Borges and Wunderlich 2008, Crespo et al. 2013, Crespo et al. 2014) or are in the process of being described (Borges et al. 2016 in press). In fact, after examining the shape and characteristics of discovery curves, Lobo and Borges (2010) clearly show that it is very likely that many new species of arthropods remain to be discovered in the Azores particularly for less studied groups in this archipelago such as Diptera and Hymenoptera. Besides purely faunistic results, the BALA data was also used to evaluate abundance, spatial variance and occupancy of arthropods (Gaston et al. 2006, Rigal et al. 2013), the effects of disturbance and biotic integrity of the native forests on arthropod assemblages (Cardoso et al. 2007, Cardoso et al. 2013, Gaspar et al. 2011, Florencio et al. 2013, Florencio et al. 2015), the extinction debt of Azorean forest specialist species (Triantis et al. 2010) and the performance of species richness estimators (Hortal et al. 2006). Moreover, such data allowed the ranking of conservation priorities for the fauna and flora of the Azores (e.g. Borges et al. 2005a, Martín et al. 2010) and allowed the estimation of extinction debt in Azores (Terzopoulou et al. 2015, Triantis et al. 2010).

During this period, two complete checklists of Azorean arthropod fauna were produced (Borges et al. 2005b, Borges et al. 2010), which included the distribution of each species per island. In this paper we compile and synthesize the faunistic results of both BALA projects, highlighting novel distribution records and presenting not only detailed distribution but also abundance data for each species, adding taxonomical and biogeographical information whenever possible. Finally, we provide a general and updated overview on the diversity of the Azorean arthropods.

## Materials and methods

***Area of study: The Azores***

**Figure 1.**
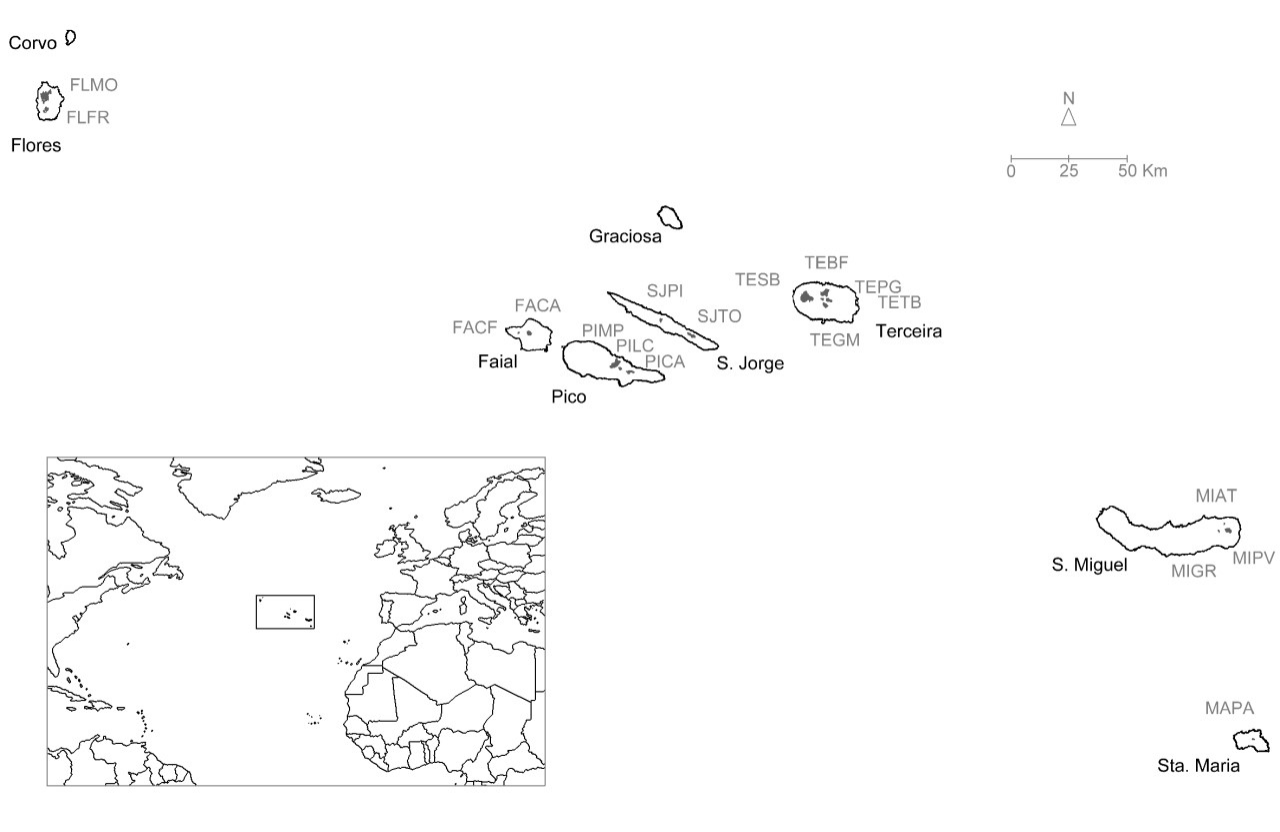
Location of the Azores and of native forest fragments in the archipelago. Codes for forest fragments as in Table 1.

The remote Azores archipelago extends for 615 km in the North Atlantic Ocean (37-40 °N, 25-31 °W), 1584 km to the east (southern Europe) and 2150 km to the west (northern America) of the nearest mainland. It comprises nine main islands and some small islets, all of volcanic origin, and is located at the triple junction of the Eurasian, African and American tectonic plates. The nine islands are divided into three groups: the western group (Corvo and Flores isls.), the central group (Faial, Pico, Graciosa, São Jorge and Terceira isls.), and the eastern group (São Miguel and Santa Maria isls) (Fig. 1). The climate is temperate and oceanic, strongly influenced by the ocean and island topography, which together produce high relative atmospheric humidity, above 95% on average on native forests.

***Sampling protocol***

Eighteen native forest fragments distributed across seven of the nine islands were sampled (Table 1; see also Gaspar et al. 2008). Graciosa and Corvo islands were excluded as they no longer present native forest. Human settlement in the Azores lead to considerable native forest destruction which has left the entire archipelago with little over 2% of the original forest cover. During the summer (June to September) 150 m long and 5 m wide transects were set up in 100 sites from 1999 to 2004 (BALA I: 18 native forest fragments) and some were sampled twice in that period totalling 123 samples; about 29 of those sites were resampled from 2010 to 2011 using the same protocol (BALA II project; 15 native forest fragments). Along each transect, arthropods from the soil (mainly epigean) and herbaceous vegetation were surveyed with pitfall traps, while arthropods from woody plants were sampled using a beating tray. Pitfall traps consisted of plastic cups with 4.2 cm diameter and 7.8 cm height. Thirty pitfall traps were set up per transect. Half of the traps were filled with a non-attractive ethylene glycol preservative solution (antifreeze solution), and the remaining with a general attractive solution, a modified version of Turquin (Turquin 1973) prepared mainly with dark beer and preservative agents. A few drops of dishwashing liquid were added to both solutions to reduce surface tension. Traps were sunk in the soil (cup rim at surface level) every 5 m along the transects, those filled with Turquin alternating with traps containing antifreeze solution. Traps were protected from rain using a plastic plate, placed about 5 cm above surface level and fixed to the ground by two pieces of wire. Accidental collection of small vertebrates and damage by rodents was prevented using a piece of plastic mesh placed on top of the trap and fixed to the ground by pieces of wire. The traps remained active in the field for two weeks.

Canopy sampling was conducted during the trapping period, when the vegetation was dry. A 5 m wide square was established every 15 m (total of 10 squares per transect). Two woody plant specimens of the most abundant species (up to three species when available) were sampled in each square. For each selected plant, a branch was chosen at random and a beating tray placed beneath. The tray consisted of a 1 m wide and 60 cm deep cloth inverted pyramid, with a plastic bag at the vertex. Five beatings were made using a stick for each plant individual sampled.

The arthropod taxa considered in this study were selected based on the availability of expert taxonomists and ability to readily separate them by morphological criteria. All Araneae, Opiliones, Pseudoscorpionida, Diplopoda, Chilopoda and Insecta (excluding Collembola, Diptera and Hymenoptera) were assigned to morphospecies through comparison with a reference collection. Various taxonomists (PAVB, ARMS, LC, PC, HE, FI, VM, MTP, JR, AB, ABS, RzS, VV, JW, JAQ, and see also Acknowledgments) checked the assignment to morphospecies, performed species identifications and supplied additional ecological information. The taxonomic nomenclature follows the most recent list of Azorean arthropods (Borges et al. 2010).

All specimens are deposited in the Entomological Collection Dalberto Teixeira Pombo at the University of the Azores (Portugal), under the curation of Paulo A. V. Borges (pborges@uac.pt).

In this contribution we list the 286 species for which we obtained an identification. The new records for each island are marked with *. For this list two families of Coleoptera were not considered since they will be presented elsewhere, Staphylinidae (Borges et al. in prep.) and Zopheridae (Borges et al. 2016). For detailed maps on the distribution of these species in Azores consult the Azores Bioportal.

All specimens were assigned a SITE CODE composed of several letters and numbers that read as follows (see Suppl. material 1 for complete data). Detailed metadata is given in Suppl. material 2):

i) the first three letters refer to island name (FLO – Flores; FAI – Faial; PIC – Pico; SJG – São Jorge; GRA – Graciosa; TER – Terceira; SMG – São Miguel; SMR – Santa Maria);

ii) the following two letters refer to fragment name (Flores: FR - Caldeiras Funda e Rasa, MA - Morro Alto e Pico da Sé; Faial: CF – Caldeira do Faial, CG – Cabeço do Fogo; Pico: CA – Caveiro, LC – Lagoa do Caiado, MP – Mistério da Prainha; São Jorge: PP – Pico Pinheiro, TO – Topo; Terceira: BF – Biscoito da Ferraria, GM – Caldeira do Guilherme Moniz, PG – Pico do Galhardo, SB –Serra de Santa Bárbara, TB – Terra Brava; São Miguel: AT – Atalhada, GR – Graminhais, PV – Pico da Vara; Santa Maria: PA – Pico Alto);

iii) the following three characters refer to the sampling transect; and

iv) the next letter refers to the sampling technique: P - pitfall, B - canopy beating; for pitfall samples (P) TU – Turquin and ET – ethylene glycol; for canopy samples (B) the next two letters refer to the plant sampled: CA = *Calluna vulgaris*, CL = *Clethra arborea*, ER = *Erica azorica*, FR = *Frangula azorica*, IL = *Ilex perado azorica*, JU = *Juniperus brevifolia*, LA = *Laurus azorica*, MC = *Morella faya*, MS = *Myrsine africana*, PI = *Picconia azorica*, PT = *Pittosporum undulatum*, VA = *Vaccinium cylindraceum*.

For the geographical location of transects within reserves (UTM coordinates) see Suppl. material 3.

Accumulation curves were obtained using the software “Species Diversity and Richness” V.4.

## Checklists

### Checklist of the Studied Azorean Arthropods

#### Animalia

#### Arthropoda

#### Arachnida

#### Pseudoscorpiones

#### Chthoniidae

#### Chthonius ischnocheles

(Hermann, 1804)

http://azoresbioportal.uac.pt/azores-species/chthonius-ischnocheles-10257/

##### Ecological interactions

###### Native status

Introduced

##### Distribution

COR; FLO*; FAI; PIC; GRA; SJG*; TER; SMG; SMR

##### Notes

Also present: MAD; CAN (Biogeographical Realm: Western Palearctic)

#### Chthonius tetrachelatus

(Preyssler, 1790)

http://azoresbioportal.uac.pt/azores-species/chthonius-tetrachelatus-10380/

##### Ecological interactions

###### Native status

Introduced

##### Distribution

COR; FLO*; FAI; PIC; GRA; SJG; TER; SMG; SMR

##### Notes

Also present: MAD; CAN (Biogeographical Realm: Western Palearctic)

#### Neobisiidae

#### Neobisium maroccanum

Beier, 1930

http://azoresbioportal.uac.pt/azores-species/neobisium-maroccanum-10482/

##### Ecological interactions

###### Native status

Introduced

##### Distribution

FLO; FAI; PIC; GRA; SJG*; TER*

##### Notes

Biogeographical Realm: Palearctic

#### Opiliones

#### Phalangiidae

#### Homalenotus coriaceus

(Simon, 1879)

http://azoresbioportal.uac.pt/azores-species/homalenotus-coriaceus-8096/

##### Ecological interactions

###### Native status

Native

##### Distribution

FLO*; FAI*; PIC*; TER*; SMG; SMR*

##### Notes

Biogeographical Realm: Palearctic

#### Leiobunum blackwalli

Meade, 1861

http://azoresbioportal.uac.pt/azores-species/leiobunum-blackwalli-7831/

##### Ecological interactions

###### Native status

Native

##### Distribution

FLO*; FAI*; PIC*; GRA; SJG*; TER*; SMG*

##### Notes

Biogeographical Realm: Western Palearctic

#### Araneae

#### Araneidae

#### Gibbaranea occidentalis

Wunderlich, 1989

http://azoresbioportal.uac.pt/azores-species/gibbaranea-occidentalis-6895/

##### Ecological interactions

###### Native status

Azores endemic

##### Distribution

FLO; FAI; PIC*; GRA; SJG*; TER*; SMG; SMR

##### Notes

Biogeographical Realm: Western Palearctic (Macaronesia)

#### Mangora acalypha

(Walckenaer, 1802)

http://azoresbioportal.uac.pt/azores-species/mangora-acalypha-7972/

##### Ecological interactions

###### Native status

Introduced

##### Distribution

FLO; FAI; PIC; GRA; SJG*; TER; SMG; SMR

##### Notes

Also present: MAD; CAN (Biogeographical Realm: Western Palearctic)

#### Clubionidae

#### Cheiracanthium erraticum

(Walckenaer, 1802)

http://azoresbioportal.uac.pt/azores-species/cheiracanthium-erraticum-6898/

##### Ecological interactions

###### Native status

Introduced

##### Distribution

FLO; FAI*; PIC*; GRA; SJG*; TER; SMG; SMR*

##### Notes

Biogeographical Realm: Palearctic

#### Cheiracanthium floresense

Wunderlich, 2008

http://azoresbioportal.uac.pt/azores-species/cheiracanthium-floresense-7719/

##### Ecological interactions

###### Native status

Azores endemic

##### Distribution

FLO*

##### Notes

Biogeographical Realm: Western Palearctic (Macaronesia)

#### Cheiracanthium jorgeense

Wunderlich, 2008

http://azoresbioportal.uac.pt/azores-species/cheiracanthium-jorgeense-7720/

##### Ecological interactions

###### Native status

Azores endemic

##### Distribution

SJG*

##### Notes

Biogeographical Realm: Western Palearctic (Macaronesia)

#### Clubiona decora

Blackwall, 1859

http://azoresbioportal.uac.pt/azores-species/clubiona-decora-7726/

##### Ecological interactions

###### Native status

Native

##### Distribution

COR; FLO*; FAI*; PIC*; GRA; SJG*; TER; SMG*; SMR*

##### Notes

Also present: MAD; CAN (Biogeographical Realm: Western Palearctic)

#### Clubiona genevensis

L. Koch, 1866

http://azoresbioportal.uac.pt/azores-species/clubiona-genevensis-7717/

##### Ecological interactions

###### Native status

Introduced

##### Distribution

FAI; PIC*; GRA; TER; SMG; SMR

##### Notes

Biogeographical Realm: Palearctic

#### Clubiona terrestris

Westring, 1851

http://azoresbioportal.uac.pt/azores-species/clubiona-terrestris-7716/

##### Ecological interactions

###### Native status

Introduced

##### Distribution

FLO*; FAI*; PIC*; GRA; TER*; SMG; SMR*

##### Notes

Biogeographical Realm: Western Palearctic

#### Dictynidae

#### Altella lucida

(Simon, 1874)

http://azoresbioportal.uac.pt/azores-species/altella-lucida-7692/

##### Ecological interactions

###### Native status

Introduced

##### Distribution

SJG*; TER

##### Notes

Biogeographical Realm: Western Palearctic

#### Emblyna acoreensis

Wunderlich, 1992

http://azoresbioportal.uac.pt/azores-species/emblyna-acoreensis-7699/

##### Ecological interactions

###### Native status

Azores endemic

##### Distribution

COR; FLO; FAI; PIC*; GRA; SJG; TER

##### Notes

Biogeographical Realm: Western Palearctic (Macaronesia)

#### Lathys dentichelis

(Simon, 1883)

http://azoresbioportal.uac.pt/azores-species/lathys-dentichelis-7083/

##### Ecological interactions

###### Native status

Native

##### Distribution

COR; FLO*; FAI*; PIC; SJG*; TER; SMG; SMR

##### Notes

Also present: MAD; CAN (Biogeographical Realm: Western Palearctic (Macaronesia))

#### Nigma puella

(Simon, 1870)

http://azoresbioportal.uac.pt/azores-species/nigma-puella-7653/

##### Ecological interactions

###### Native status

Introduced

##### Distribution

COR; FLO; FAI; PIC*; GRA; SJG*; TER; SMG; SMR

##### Notes

Also present: MAD; CAN (Biogeographical Realm: Western Palearctic (Macaronesia))

#### Dysderidae

#### Dysdera crocata

C. L. Koch, 1838

http://azoresbioportal.uac.pt/azores-species/dysdera-crocata-7212/

##### Ecological interactions

###### Native status

Introduced

##### Distribution

COR; FLO; FAI; PIC; GRA; SJG*; TER; SMG; SMR

##### Notes

Also present: MAD (Biogeographical Realm: Cosmopolitan)

#### Linyphiidae

#### Acorigone acoreensis

(Wunderlich, 1992)

http://azoresbioportal.uac.pt/azores-species/acorigone-acoreensis-7081/

##### Ecological interactions

###### Native status

Azores endemic

##### Distribution

FLO*; FAI*; PIC*; SJG*; TER; SMG*; SMR*

##### Notes

Biogeographical Realm: Western Palearctic (Macaronesia)

#### Acorigone zebraneus

Wunderlich, 2008

http://azoresbioportal.uac.pt/azores-species/acorigone-zebraneus-7753/

##### Ecological interactions

###### Native status

Azores endemic

##### Distribution

SJG*

##### Notes

Biogeographical Realm: Western Palearctic (Macaronesia)

#### Agyneta decora

(O. P.-Cambridge, 1871)

http://azoresbioportal.uac.pt/azores-species/agyneta-decora-7739/

##### Ecological interactions

###### Native status

Introduced

##### Distribution

FLO*; SJG*; TER

##### Notes

Biogeographical Realm: Palearctic

#### Agyneta depigmentata

Wunderlich, 2008

http://azoresbioportal.uac.pt/azores-species/agyneta-depigmentata-6947/

##### Ecological interactions

###### Native status

Azores endemic

##### Distribution

FLO*

##### Notes

Biogeographical Realm: Western Palearctic (Macaronesia)

#### Agyneta rugosa

Wunderlich, 1992

http://azoresbioportal.uac.pt/azores-species/agyneta-rugosa-7740/

##### Ecological interactions

###### Native status

Azores endemic

##### Distribution

FAI*; SJG; SMG

##### Notes

Biogeographical Realm: Western Palearctic (Macaronesia)

#### Canariphantes acoreensis

(Wunderlich, 1992)

http://azoresbioportal.uac.pt/azores-species/canariphantes-acoreensis-12410/

##### Ecological interactions

###### Native status

Azores endemic

##### Distribution

FAI; PIC; SJG*; TER

##### Notes

Biogeographical Realm: Western Palearctic (Macaronesia)

#### Canariphantes junipericola

Crespo & Bosmans, 2014

http://azoresbioportal.uac.pt/azores-species/canariphantes-junipericola-12407/

##### Ecological interactions

###### Native status

Azores endemic

##### Distribution

FLO*

##### Notes

Biogeographical Realm: Western Palearctic (Macaronesia)

#### Canariphantes relictus

Crespo & Bosmans, 2014

http://azoresbioportal.uac.pt/azores-species/canariphantes-relictus-12412/

##### Ecological interactions

###### Native status

Azores endemic

##### Distribution

SMR*

##### Notes

Biogeographical Realm: Western Palearctic (Macaronesia)

#### Erigone atra

Blackwall, 1833

http://azoresbioportal.uac.pt/azores-species/erigone-atra-7096/

##### Ecological interactions

###### Native status

Introduced

##### Distribution

COR; FLO; FAI; PIC; GRA; SJG*; TER; SMG; SMR

##### Notes

Also present: MAD; CAN (Biogeographical Realm: Holarctic)

#### Erigone autumnalis

Emerton, 1882

http://azoresbioportal.uac.pt/azores-species/erigone-autumnalis-7758/

##### Ecological interactions

###### Native status

Introduced

##### Distribution

FLO*; FAI; PIC; GRA; SJG*; TER; SMG; SMR

##### Notes

Also present: CAN (Biogeographical Realm: Nearctic)

#### Erigone dentipalpis

(Wider, 1834)

http://azoresbioportal.uac.pt/azores-species/erigone-dentipalpis-7759/

##### Ecological interactions

###### Native status

Introduced

##### Distribution

FLO*; FAI; PIC; GRA; SJG; TER; SMG; SMR

##### Notes

Also present: MAD; CAN (Biogeographical Realm: Holarctic)

#### Lessertia dentichelis

(Simon, 1884)

http://azoresbioportal.uac.pt/azores-species/lessertia-dentichelis-7773/

##### Ecological interactions

###### Native status

Introduced

##### Distribution

SMG*

##### Notes

Also present: MAD; CAN (Biogeographical Realm: Western Palearctic)

#### Meioneta fuscipalpa

(C. L. Koch, 1836)

http://azoresbioportal.uac.pt/azores-species/meioneta-fuscipalpa-7742/

##### Ecological interactions

###### Native status

Introduced

##### Distribution

COR; FLO; FAI; PIC; GRA; SJG; TER; SMG; SMR

##### Notes

Also present: MAD (Biogeographical Realm: Palearctic)

#### Mermessus bryantae

(Ivie & Barrows, 1935)

http://azoresbioportal.uac.pt/azores-species/mermessus-bryantae-7755/

##### Ecological interactions

###### Native status

Introduced

##### Distribution

FAI; PIC*; GRA; SJG*; TER; SMG

##### Notes

Biogeographical Realm: Nearctic

#### Mermessus fradeorum

(Berland, 1932)

http://azoresbioportal.uac.pt/azores-species/mermessus-fradeorum-7756/

##### Ecological interactions

###### Native status

Introduced

##### Distribution

FLO; FAI; PIC; GRA; TER; SMG; SMR

##### Notes

Biogeographical Realm: Cosmopolitan

#### Mermessus trilobatus

(Emerton, 1882)

http://azoresbioportal.uac.pt/azores-species/mermessus-trilobatus-7757/

##### Ecological interactions

###### Native status

Introduced

##### Distribution

SJG*; TER*; SMG*

##### Notes

Biogeographical Realm: Holarctic

#### Microlinyphia johnsoni

(Blackwall, 1859)

http://azoresbioportal.uac.pt/azores-species/microlinyphia-johnsoni-7150/

##### Ecological interactions

###### Native status

Introduced

##### Distribution

FAI; PIC; SJG; TER; SMG

##### Notes

Also present: MAD; CAN (Biogeographical Realm: Western Palearctic (Macaronesia))

#### Minicia floresensis

Wunderlich, 1992

http://azoresbioportal.uac.pt/azores-species/minicia-floresensis-7215/

##### Ecological interactions

###### Native status

Azores endemic

##### Distribution

FLO; PIC; SJG*; TER*; SMG*

##### Notes

Biogeographical Realm: Western Palearctic (Macaronesia)

#### Neriene clathrata

(Sundevall, 1830)

http://azoresbioportal.uac.pt/azores-species/neriene-clathrata-7772/

##### Ecological interactions

###### Native status

Introduced

##### Distribution

FAI; SJG; TER*; SMG

##### Notes

Biogeographical Realm: Holarctic

#### Oedothorax fuscus

(Blackwall, 1834)

http://azoresbioportal.uac.pt/azores-species/oedothorax-fuscus-7763/

##### Ecological interactions

###### Native status

Introduced

##### Distribution

COR; FLO*; FAI*; PIC; GRA; SJG*; TER; SMG*; SMR

##### Notes

Biogeographical Realm: Western Palearctic; Mediterranean

#### Palliduphantes schmitzi

(Kulczynski, 1899)

http://azoresbioportal.uac.pt/azores-species/palliduphantes-schmitzi-7743/

##### Ecological interactions

###### Native status

Native

##### Distribution

COR; FLO; FAI; PIC; GRA; SJG*; TER; SMG; SMR*

##### Notes

Also present: MAD (Biogeographical Realm: Western Palearctic (Macaronesia))

#### Pelecopsis parallela

(Wider, 1834)

http://azoresbioportal.uac.pt/azores-species/pelecopsis-parallela-7769/

##### Ecological interactions

###### Native status

Introduced

##### Distribution

FAI*; PIC; SJG; TER; SMG

##### Notes

Biogeographical Realm: Palearctic

#### Porrhomma borgesi

Wunderlich, 2008

http://azoresbioportal.uac.pt/azores-species/porrhomma-borgesi-7734/

##### Ecological interactions

###### Native status

Azores endemic

##### Distribution

PIC*; TER*; SMG*

##### Notes

Biogeographical Realm: Western Palearctic (Macaronesia)

#### Prinerigone vagans

(Audouin, 1826)

http://azoresbioportal.uac.pt/azores-species/prinerigone-vagans-7761/

##### Ecological interactions

###### Native status

Introduced

##### Distribution

FLO; PIC; GRA; TER; SMG; SMR

##### Notes

Also present: MAD; CAN (Biogeographical Realm: Palearctic)

#### Savigniorrhipis acoreensis

Wunderlich, 1992

http://azoresbioportal.uac.pt/azores-species/savigniorrhipis-acoreensis-7160/

##### Ecological interactions

###### Native status

Azores endemic

##### Distribution

FLO*; FAI; PIC; SJG*; TER; SMG; SMR

##### Notes

Biogeographical Realm: Western Palearctic (Macaronesia)

#### Savigniorrhipis topographicus

Crespo, 2013

http://azoresbioportal.uac.pt/azores-species/savigniorrhipis-topographicus-7061/

##### Ecological interactions

###### Native status

Azores endemic

##### Distribution

SJG*

##### Notes

Biogeographical Realm: Western Palearctic (Macaronesia)

#### Tenuiphantes miguelensis

(Wunderlich, 1992)

http://azoresbioportal.uac.pt/azores-species/tenuiphantes-miguelensis-7084/

##### Ecological interactions

###### Native status

Native

##### Distribution

FLO*; FAI*; PIC*; GRA; SJG*; TER; SMG; SMR*

##### Notes

Also present: MAD (Biogeographical Realm: Western Palearctic (Macaronesia))

#### Tenuiphantes tenuis

(Blackwall, 1852)

http://azoresbioportal.uac.pt/azores-species/tenuiphantes-tenuis-7161/

##### Ecological interactions

###### Native status

Introduced

##### Distribution

COR; FLO; FAI; PIC; GRA; SJG; TER; SMG; SMR

##### Notes

Also present: MAD; CAN (Biogeographical Realm: Western Palearctic; Mediterranean)

#### Walckenaeria grandis

(Wunderlich, 1992)

http://azoresbioportal.uac.pt/azores-species/walckenaeria-grandis-7213/

##### Ecological interactions

###### Native status

Azores endemic

##### Distribution

FLO*; PIC*; SJG*; TER; SMG

##### Notes

Biogeographical Realm: Western Palearctic (Macaronesia)

#### Lycosidae

#### Pardosa acorensis

Simon, 1883

http://azoresbioportal.uac.pt/azores-species/pardosa-acorensis-7712/

##### Ecological interactions

###### Native status

Azores endemic

##### Distribution

COR; FLO; FAI; PIC; GRA; SJG*; TER; SMG; SMR

##### Notes

Biogeographical Realm: Western Palearctic (Macaronesia)

#### Mimetidae

#### Ero furcata

(Villers, 1789)

http://azoresbioportal.uac.pt/azores-species/ero-furcata-7752/

##### Ecological interactions

###### Native status

Introduced

##### Distribution

COR; FLO*; FAI*; PIC; GRA; SJG*; TER; SMG*; SMR

##### Notes

Also present: MAD; CAN (Biogeographical Realm: Palearctic)

#### Oecobiidae

#### Oecobius navus

Blackwall, 1859

http://azoresbioportal.uac.pt/azores-species/oecobius-navus-7963/

##### Ecological interactions

###### Native status

Introduced

##### Distribution

FAI; PIC; SJG; TER; SMG; SMR

##### Notes

Also present: MAD; CAN; CVP (Biogeographical Realm: Cosmopolitan)

#### Oonopidae

#### Orchestina furcillata

Wunderlich, 2008

http://azoresbioportal.uac.pt/azores-species/orchestina-furcillata-7958/

##### Ecological interactions

###### Native status

Azores endemic

##### Distribution

SMG*

##### Notes

Biogeographical Realm: Western Palearctic (Macaronesia)

#### Pisauridae

#### Pisaura acoreensis

Wunderlich, 1992

http://azoresbioportal.uac.pt/azores-species/pisaura-acoreensis-7082/

##### Ecological interactions

###### Native status

Azores endemic

##### Distribution

FLO; FAI; PIC*; GRA; SJG*; TER; SMG; SMR

##### Notes

Biogeographical Realm: Western Palearctic (Macaronesia)

#### Salticidae

#### Macaroeris cata

(Blackwall, 1867)

http://azoresbioportal.uac.pt/azores-species/macaroeris-cata-7152/

##### Ecological interactions

###### Native status

Native

##### Distribution

COR; FLO; FAI; PIC*; GRA; SJG*; TER; SMG; SMR

##### Notes

Also present: MAD; CAN (Biogeographical Realm: Western Palearctic (Macaronesia); Romania)

#### Macaroeris diligens

(Blackwall, 1867)

http://azoresbioportal.uac.pt/azores-species/macaroeris-diligens-7736/

##### Ecological interactions

###### Native status

Native

##### Distribution

COR; FAI; TER; SMG; SMR*

##### Notes

Also present: MAD; CAN (Biogeographical Realm: Western Palearctic (Macaronesia))

#### Neon acoreensis

Wunderlich, 2008

http://azoresbioportal.uac.pt/azores-species/neon-acoreensis-7790/

##### Ecological interactions

###### Native status

Azores endemic

##### Distribution

FLO; FAI; PIC*; SJG*; TER; SMG; SMR

##### Notes

Biogeographical Realm: Western Palearctic (Macaronesia)

#### Pseudeuophrys vafra

(Blackwall, 1867)

http://azoresbioportal.uac.pt/azores-species/pseudeuophrys-vafra-7701/

##### Ecological interactions

###### Native status

Introduced

##### Distribution

COR; FLO; FAI; PIC; GRA; SJG; TER; SMG; SMR

##### Notes

Also present: MAD (Biogeographical Realm: Palearctic)

#### Tetragnathidae

#### Metellina merianae

(Scopoli, 1763)

http://azoresbioportal.uac.pt/azores-species/metellina-merianae-7965/

##### Ecological interactions

###### Native status

Introduced

##### Distribution

FLO*; FAI; PIC; GRA; SJG; TER; SMG; SMR

##### Notes

Also present: MAD (Biogeographical Realm: Palearctic)

#### Sancus acoreensis

(Wunderlich, 1992)

http://azoresbioportal.uac.pt/azores-species/sancus-acoreensis-7971/

##### Ecological interactions

###### Native status

Azores endemic

##### Distribution

FLO; FAI; PIC; SJG*; TER; SMG; SMR

##### Notes

Biogeographical Realm: Western Palearctic (Macaronesia)

#### Theridiidae

#### Cryptachaea blattea

(Urquhart, 1886)

http://azoresbioportal.uac.pt/azores-species/cryptachaea-blattea-7774/

##### Ecological interactions

###### Native status

Introduced

##### Distribution

COR; FLO; FAI; PIC; GRA; TER; SMG; SMR

##### Notes

Also present: CAN (Biogeographical Realm: Nearctic)

#### Lasaeola oceanica

Simon, 1883

http://azoresbioportal.uac.pt/azores-species/lasaeola-oceanica-7751/

##### Ecological interactions

###### Native status

Azores endemic

##### Distribution

COR; FLO; FAI*; PIC*; GRA; SJG*; TER; SMG; SMR

##### Notes

Biogeographical Realm: Western Palearctic (Macaronesia)

#### Neottiura bimaculata

(Linnaeus, 1767)

http://azoresbioportal.uac.pt/azores-species/neottiura-bimaculata-7778/

##### Ecological interactions

###### Native status

Introduced

##### Distribution

PIC; GRA; SJG; TER; SMG; SMR

##### Notes

Biogeographical Realm: Holarctic

#### Rhomphaea nasica

(Simon, 1873)

http://azoresbioportal.uac.pt/azores-species/rhomphaea-nasica-7766/

##### Ecological interactions

###### Native status

Introduced

##### Distribution

FLO; PIC; GRA; TER; SMG

##### Notes

Also present: MAD (Biogeographical Realm: Palearctic)

#### Rugathodes acoreensis

Wunderlich, 1992

http://azoresbioportal.uac.pt/azores-species/rugathodes-acoreensis-7698/

##### Ecological interactions

###### Native status

Azores endemic

##### Distribution

FLO; FAI; PIC; GRA; SJG*; TER; SMG; SMR

##### Notes

Biogeographical Realm: Western Palearctic (Macaronesia)

#### Steatoda grossa

(C. L. Koch, 1838)

http://azoresbioportal.uac.pt/azores-species/steatoda-grossa-7691/

##### Ecological interactions

###### Native status

Introduced

##### Distribution

COR; FLO; FAI; PIC; GRA; SJG; TER; SMG; SMR

##### Notes

Also present: MAD; CAN (Biogeographical Realm: Cosmopolitan)

#### Theridion melanurum

Hahn, 1831

http://azoresbioportal.uac.pt/azores-species/theridion-melanurum-9697/

##### Ecological interactions

###### Native status

Introduced

##### Distribution

PIC*; SMG*; SMR*

##### Notes

Also present: MAD (Biogeographical Realm: Holarctic)

#### Theridion musivivum

Schmidt, 1956

http://azoresbioportal.uac.pt/azores-species/theridion-musivivum-7703/

##### Ecological interactions

###### Native status

Native

##### Distribution

COR; FLO; FAI; PIC*; GRA; TER; SMG; SMR

##### Notes

Also present: MAD; CAN; CVP (Biogeographical Realm: Western Palearctic (Macaronesia))

#### Thomisidae

#### Xysticus cor

Canestrini, 1873

http://azoresbioportal.uac.pt/azores-species/xysticus-cor-7922/

##### Ecological interactions

###### Native status

Native

##### Distribution

COR; FLO; FAI*; PIC; GRA; SJG*; TER; SMG; SMR*

##### Notes

Biogeographical Realm: Palearctic

#### Xysticus nubilus

Simon, 1875

http://azoresbioportal.uac.pt/azores-species/xysticus-nubilus-7737/

##### Ecological interactions

###### Native status

Introduced

##### Distribution

FLO*; FAI; PIC; GRA; SJG*; TER; SMG; SMR

##### Notes

Also present: MAD; CAN (Biogeographical Realm: Palearctic)

#### Zodariidae

#### Zodarion atlanticum

Pekár & Cardoso, 2006

http://azoresbioportal.uac.pt/azores-species/zodarion-atlanticum-7786/

##### Ecological interactions

###### Native status

Introduced

##### Distribution

FAI; PIC; GRA; TER*; SMG

##### Notes

Biogeographical Realm: Palearctic

#### Diplopoda

#### Polydesmida

#### Paradoxosomatidae

#### Oxidus gracilis

(C. L. Koch, 1847)

http://azoresbioportal.uac.pt/azores-species/oxidus-gracilis-8134/

##### Ecological interactions

###### Native status

Introduced

##### Distribution

COR; FLO*; FAI*; PIC; GRA; TER*; SMG; SMR*

##### Notes

Also present: MAD; CAN (Biogeographical Realm: Afro-tropical; Australian; Eastern Palearctic; Nearctic; Neotropical; Oriental)

#### Polydesmidae

#### Brachydesmus superus

Latzel, 1884

http://azoresbioportal.uac.pt/azores-species/brachydesmus-superus-8136/

##### Ecological interactions

###### Native status

Introduced

##### Distribution

FLO*; FAI; PIC; SJG; TER*; SMG; SMR

##### Notes

Also present: MAD; CAN; CVP (Biogeographical Realm: Australian; Eastern Palearctic; Nearctic; North Africa)

#### Polydesmus coriaceus

Porat, 1871

http://azoresbioportal.uac.pt/azores-species/polydesmus-coriaceus-8146/

##### Ecological interactions

###### Native status

Introduced

##### Distribution

COR; FLO*; FAI; PIC; GRA; SJG*; TER; SMG; SMR

##### Notes

Also present: CAN (Biogeographical Realm: Western Palearctic)

#### Julida

#### Blaniulidae

#### Blaniulus guttulatus

(Fabricius, 1798)

http://azoresbioportal.uac.pt/azores-species/blaniulus-guttulatus-8151/

##### Ecological interactions

###### Native status

Introduced

##### Distribution

COR; FLO; FAI; PIC; GRA; SJG; TER; SMG; SMR*

##### Notes

Also present: MAD (Biogeographical Realm: Australian; Eastern Palearctic; Nearctic)

#### Choneiulus palmatus

(Nemec, 1895)

http://azoresbioportal.uac.pt/azores-species/choneiulus-palmatus-8152/

##### Ecological interactions

###### Native status

Introduced

##### Distribution

FLO*; PIC*; GRA; SJG*; TER; SMG; SMR*

##### Notes

Also present: MAD; CAN (Biogeographical Realm: Nearctic)

#### Nopoiulus kochii

(Gervais, 1847)

http://azoresbioportal.uac.pt/azores-species/nopoiulus-kochii-8153/

##### Ecological interactions

###### Native status

Introduced

##### Distribution

FLO*; GRA; SMG; SMR*

##### Notes

Also present: MAD; CAN (Biogeographical Realm: Australian; Near East; Nearctic; Neotropical)

#### Proteroiulus fuscus

(Am Stein, 1857)

http://azoresbioportal.uac.pt/azores-species/proteroiulus-fuscus-8154/

##### Ecological interactions

###### Native status

Introduced

##### Distribution

FLO*; FAI; TER*; SMG; SMR*

##### Notes

Also present: MAD; CAN (Biogeographical Realm: Nearctic)

#### Julidae

#### Brachyiulus pusillus

(Leach, 1814)

http://azoresbioportal.uac.pt/azores-species/brachyiulus-pusillus-8156/

##### Ecological interactions

###### Native status

Introduced

##### Distribution

FLO; FAI; GRA; TER; SMG; SMR

##### Notes

Also present: MAD; CAN (Biogeographical Realm: Afro-tropical; Australian; Nearctic)

#### Cylindroiulus latestriatus

(Curtis, 1845)

http://azoresbioportal.uac.pt/azores-species/cylindroiulus-latestriatus-8159/

##### Ecological interactions

###### Native status

Introduced

##### Distribution

COR; FLO*; FAI; SMG; SMR

##### Notes

Biogeographical Realm: Afro-tropical; Australian; Nearctic; Oriental

#### Cylindroiulus propinquus

(Porat, 1870)

http://azoresbioportal.uac.pt/azores-species/cylindroiulus-propinquus-8161/

##### Ecological interactions

###### Native status

Introduced

##### Distribution

COR; FLO; FAI; PIC; GRA; SJG*; TER; SMG; SMR

##### Notes

Biogeographical Realm: Palearctic

#### Ommatoiulus moreletii

(Lucas, 1860)

http://azoresbioportal.uac.pt/azores-species/ommatoiulus-moreletii-6874/

##### Ecological interactions

###### Native status

Introduced

##### Distribution

COR; FLO; FAI; PIC; GRA; SJG; TER; SMG; SMR

##### Notes

Also present: MAD; CAN (Biogeographical Realm: Afro-tropical; Australian)

#### Chordeumatida

#### Haplobainosomatidae

#### Haplobainosoma lusitanum

Verhoeff, 1900

http://azoresbioportal.uac.pt/azores-species/haplobainosoma-lusitanum-8162/

##### Ecological interactions

###### Native status

Introduced

##### Distribution

FAI*; PIC; TER*; SMG*; SMR*

##### Notes

Biogeographical Realm: Palearctic

#### Chilopoda

#### Lithobiomorpha

#### Lithobiidae

#### Lithobius pilicornis pilicornis

Newport, 1844

http://azoresbioportal.uac.pt/azores-species/lithobius-pilicornis-pilicornis-13445/

##### Ecological interactions

###### Native status

Native

##### Distribution

COR; FLO; FAI; PIC; GRA; SJG; TER; SMG; SMR

##### Notes

Also present: MAD; CAN (Biogeographical Realm: Afro-tropical)

#### Scolopendromorpha

#### Cryptopidae

#### Cryptops hortensis

(Donovan, 1810)

http://azoresbioportal.uac.pt/azores-species/cryptops-hortensis-8171/

##### Ecological interactions

###### Native status

Native

##### Distribution

COR; FLO; FAI*; PIC; GRA; SJG; TER*; SMG

##### Notes

Also present: MAD; CAN (Biogeographical Realm: Western Palearctic)

#### Geophilomorpha

#### Geophilidae

#### Geophilus truncorum

Bergsoe & Meinert, 1866

http://azoresbioportal.uac.pt/azores-species/geophilus-truncorum-8174/

##### Ecological interactions

###### Native status

Native

##### Distribution

FLO; FAI; PIC*; GRA; SJG*; TER*; SMG*; SMR

##### Notes

Also present: MAD (Biogeographical Realm: Western Palearctic)

#### Linotaeniidae

#### Strigamia crassipes

(C. L. Koch, 1835)

http://azoresbioportal.uac.pt/azores-species/strigamia-crassipes-8177/

##### Ecological interactions

###### Native status

Native

##### Distribution

FLO*; TER*; SMG*

##### Notes

Biogeographical Realm: Western Palearctic

#### Insecta

#### Microcoryphia

#### Machilidae

#### Dilta saxicola

(Womersley, 1930)

http://azoresbioportal.uac.pt/azores-species/dilta-saxicola-8352/

##### Ecological interactions

###### Native status

Native

##### Distribution

COR; FLO; FAI; PIC; GRA; SJG; TER; SMG; SMR

##### Notes

Biogeographical Realm: Easternern Palearctic

#### Trigoniophthalmus borgesi

Mendes, Gaju, Bach & Molero, 2000

http://azoresbioportal.uac.pt/azores-species/trigoniophthalmus-borgesi-8350/

##### Ecological interactions

###### Native status

Azores endemic

##### Distribution

FAI*; PIC*; SJG*; TER; SMG*; SMR

##### Notes

Biogeographical Realm: Western Palearctic (Macaronesia)

#### Ephemeroptera

#### Baetidae

#### Cloeon dipterum

Linnaeus, 1761

http://azoresbioportal.uac.pt/azores-species/cloeon-dipterum-8286/

##### Ecological interactions

###### Native status

Native

##### Distribution

FAI; PIC; GRA; SJG; TER; SMG; SMR

##### Notes

Also present: MAD; CAN (Biogeographical Realm: Holarctic)

#### Blattaria

#### Polyphagidae

#### Zetha vestita

(Brullé, 1838)

http://azoresbioportal.uac.pt/azores-species/zetha-vestita-7095/

##### Ecological interactions

###### Native status

Native

##### Distribution

FLO*; FAI; PIC*; SJG; TER; SMG; SMR*

##### Notes

Also present: MAD; CAN; CVP (Biogeographical Realm: Palearctic)

#### Orthoptera

#### Conocephalidae

#### Conocephalus chavesi

(Bolivar, 1905)

http://azoresbioportal.uac.pt/azores-species/conocephalus-chavesi-8303/

##### Ecological interactions

###### Native status

Azores endemic

##### Distribution

PIC; TER; SMG

##### Notes

Biogeographical Realm: Western Palearctic (Macaronesia)

#### Gryllidae

#### Gryllus bimaculatus

De Geer, 1773

http://azoresbioportal.uac.pt/azores-species/gryllus-bimaculatus-8305/

##### Ecological interactions

###### Native status

Introduced

##### Distribution

COR; FLO; FAI; PIC; GRA; SJG; TER; SMG; SMR

##### Notes

Also present: MAD; CAN; CVP (Biogeographical Realm: Afro-tropical; Eastern Palearctic; Near East; North Africa; Oriental)

#### Dermaptera

#### Anisolabididae

#### Euborellia annulipes

(Lucas, 1847)

http://azoresbioportal.uac.pt/azores-species/euborellia-annulipes-8315/

##### Ecological interactions

###### Native status

Introduced

##### Distribution

COR; FLO; FAI; PIC; GRA; SJG; TER; SMG; SMR

##### Notes

Also present: MAD; CAN; CVP (Biogeographical Realm: Cosmopolitan)

#### Forficulidae

#### Forficula auricularia

Linnaeus, 1758

http://azoresbioportal.uac.pt/azores-species/forficula-auricularia-8316/

##### Ecological interactions

###### Native status

Introduced

##### Distribution

COR; FLO; FAI; PIC; GRA; SJG; TER; SMG; SMR

##### Notes

Also present: MAD; CAN (Biogeographical Realm: Holarctic)

#### Psocoptera

#### Caeciliusidae

#### Valenzuela burmeisteri

(Brauer, 1876)

http://azoresbioportal.uac.pt/azores-species/valenzuela-burmeisteri-7098/

##### Ecological interactions

###### Native status

Native

##### Distribution

FLO*; FAI*; SJG*; TER*; SMG*; SMR*

##### Notes

Also present: MAD; CAN (Biogeographical Realm: Holarctic)

#### Valenzuela flavidus

(Stephens, 1836)

http://azoresbioportal.uac.pt/azores-species/valenzuela-flavidus-8351/

##### Ecological interactions

###### Native status

Native

##### Distribution

COR; FLO; FAI*; PIC*; GRA; SJG*; TER; SMG; SMR

##### Notes

Also present: MAD; CAN (Biogeographical Realm: Holarctic)

#### Ectopsocidae

#### Ectopsocus briggsi

McLachlan, 1899

http://azoresbioportal.uac.pt/azores-species/ectopsocus-briggsi-8349/

##### Ecological interactions

###### Native status

Introduced

##### Distribution

COR; FLO; FAI*; PIC; GRA; SJG; TER; SMG; SMR

##### Notes

Also present: MAD; CAN (Biogeographical Realm: Cosmopolitan)

#### Ectopsocus pumilis

(Banks, 1920)

http://azoresbioportal.uac.pt/azores-species/ectopsocus-pumilis-8331/

##### Ecological interactions

###### Native status

Introduced

##### Distribution

SMG; SMR*

##### Notes

Biogeographical Realm: Cosmopolitan

#### Ectopsocus strauchi

Enderlein, 1906

http://azoresbioportal.uac.pt/azores-species/ectopsocus-strauchi-8333/

##### Ecological interactions

###### Native status

Native

##### Distribution

COR; FLO; FAI*; PIC*; GRA; TER; SMG; SMR

##### Notes

Also present: MAD; CAN; CVP (Biogeographical Realm: Palearctic)

#### Elipsocidae

#### Elipsocus azoricus

Meinander, 1975

http://azoresbioportal.uac.pt/azores-species/elipsocus-azoricus-7156/

##### Ecological interactions

###### Native status

Azores endemic

##### Distribution

COR; FLO*; FAI*; PIC*; GRA; SJG*; TER*; SMG; SMR

##### Notes

Biogeographical Realm: Western Palearctic (Macaronesia)

#### Elipsocus brincki

Badonnel, 1963

http://azoresbioportal.uac.pt/azores-species/elipsocus-brincki-8348/

##### Ecological interactions

###### Native status

Azores endemic

##### Distribution

COR; FLO*; FAI*; PIC*; GRA; SJG; TER*; SMG; SMR*

##### Notes

Biogeographical Realm: Western Palearctic (Macaronesia)

#### Epipsocidae

#### Bertkauia lucifuga

(Rambur, 1842)

http://azoresbioportal.uac.pt/azores-species/bertkauia-lucifuga-8346/

##### Ecological interactions

###### Native status

Native

##### Distribution

FAI*; TER; SMG

##### Notes

Also present: MAD (Biogeographical Realm: Western Palearctic)

#### Lachesillidae

#### Lachesilla greeni

(Pearman, 1933)

http://azoresbioportal.uac.pt/azores-species/lachesilla-greeni-8334/

##### Ecological interactions

###### Native status

Introduced

##### Distribution

TER*; SMG; SMR*

##### Notes

Also present: MAD (Biogeographical Realm: Western Palearctic)

#### Peripsocidae

#### Peripsocus milleri

(Tillyard, 1923)

http://azoresbioportal.uac.pt/azores-species/peripsocus-milleri-8341/

##### Ecological interactions

###### Native status

Native

##### Distribution

FAI*; SJG*; TER*; SMG

##### Notes

Also present: MAD; CAN (Biogeographical Realm: Cosmopolitan)

#### Peripsocus phaeopterus

(Stephens, 1836)

http://azoresbioportal.uac.pt/azores-species/peripsocus-phaeopterus-8342/

##### Ecological interactions

###### Native status

Native

##### Distribution

PIC*; SJG*; TER*; SMG; SMR

##### Notes

Also present: CAN (Biogeographical Realm: Palearctic)

#### Peripsocus subfasciatus

(Rambur, 1842)

http://azoresbioportal.uac.pt/azores-species/peripsocus-subfasciatus-8343/

##### Ecological interactions

###### Native status

Native

##### Distribution

FAI*; TER*; SMG; SMR

##### Notes

Biogeographical Realm: Holarctic

#### Psocidae

#### Atlantopsocus adustus

(Hagen, 1865)

http://azoresbioportal.uac.pt/azores-species/atlantopsocus-adustus-6897/

##### Ecological interactions

###### Native status

Native

##### Distribution

FLO*; FAI*; PIC*; GRA; TER; SMG; SMR

##### Notes

Also present: MAD; CAN (Biogeographical Realm: Western Palearctic (Macaronesia))

#### Trichopsocidae

#### Trichopsocus clarus

(Banks, 1908)

http://azoresbioportal.uac.pt/azores-species/trichopsocus-clarus-7158/

##### Ecological interactions

###### Native status

Native

##### Distribution

COR; FLO; FAI*; PIC*; GRA; SJG; TER; SMG; SMR

##### Notes

Also present: MAD; CAN (Biogeographical Realm: Cosmopolitan)

#### Trogiidae

#### Lepinotus reticulatus

Enderlein, 1905

http://azoresbioportal.uac.pt/azores-species/lepinotus-reticulatus-8362/

##### Ecological interactions

###### Native status

Introduced

##### Distribution

TER*

##### Notes

Also present: MAD; CAN (Biogeographical Realm: Cosmopolitan)

#### Hemiptera

#### Anthocoridae

#### Brachysteles parvicornis

(A. Costa, 1847)

http://azoresbioportal.uac.pt/azores-species/brachysteles-parvicornis-7079/

##### Ecological interactions

###### Native status

Native

##### Distribution

PIC*; GRA; TER*; SMG*; SMR

##### Notes

Also present: CAN (Biogeographical Realm: Holactic; Afro-tropical; Northern Asia (except China))

#### Buchananiella continua

(White, 1880)

http://azoresbioportal.uac.pt/azores-species/buchananiella-continua-7214/

##### Ecological interactions

###### Native status

Introduced

##### Distribution

FLO; FAI; PIC; SJG*; TER; SMG; SMR*

##### Notes

Also present: MAD; CAN (Biogeographical Realm: Afro-tropical; Australian; Neotropical)

#### Orius laevigatus laevigatus

(Fieber, 1860)

http://azoresbioportal.uac.pt/azores-species/orius-laevigatus-laevigatus-13520/

##### Ecological interactions

###### Native status

Native

##### Distribution

FLO; FAI; PIC; GRA; SJG; TER; SMG; SMR

##### Notes

Also present: CAN (Biogeographical Realm: Oriental)

#### Aphididae

#### Acyrthosiphon pisum

(Harris, 1776)

http://azoresbioportal.uac.pt/azores-species/acyrthosiphon-pisum-8553/

##### Ecological interactions

###### Native status

Native

##### Distribution

FLO*; FAI; PIC; GRA; SJG; TER; SMG; SMR

##### Notes

Also present: MAD (Biogeographical Realm: Cosmopolitan)

#### Amphorophora rubi

(Kaltenbach, 1843)

http://azoresbioportal.uac.pt/azores-species/amphorophora-rubi-8560/

##### Ecological interactions

###### Native status

Native

##### Distribution

FLO*; GRA; TER

##### Notes

Also present: MAD; CAN (Biogeographical Realm: Australian; Eastern Palearctic; Near East; Nearctic; North Africa)

#### Aphis craccivora

Koch, 1854

http://azoresbioportal.uac.pt/azores-species/aphis-craccivora-8555/

##### Ecological interactions

###### Native status

Native

##### Distribution

COR; FLO; FAI; PIC; TER; SMG; SMR

##### Notes

Also present: MAD; CAN; CVP (Biogeographical Realm: Cosmopolitan)

#### Aulacorthum solani

(Kaltenbach, 1843)

http://azoresbioportal.uac.pt/azores-species/aulacorthum-solani-8576/

##### Ecological interactions

###### Native status

Native

##### Distribution

FLO; FAI*; TER; SMG; SMR

##### Notes

Also present: MAD; CAN; CVP (Biogeographical Realm: Cosmopolitan)

#### Cavariella aegopodii

(Scopoli, 1763)

http://azoresbioportal.uac.pt/azores-species/cavariella-aegopodii-8590/

##### Ecological interactions

###### Native status

Introduced

##### Distribution

FLO; SJG*; TER; SMG; SMR

##### Notes

Biogeographical Realm: Cosmopolitan

#### Dysaphis plantaginea

(Passerini, 1860)

http://azoresbioportal.uac.pt/azores-species/dysaphis-plantaginea-8602/

##### Ecological interactions

###### Native status

Introduced

##### Distribution

FLO; FAI; GRA; TER; SMG; SMR

##### Notes

Also present: MAD; CAN (Biogeographical Realm: Cosmopolitan)

#### Longiunguis luzulella

Hille Ris Lambers, 1947

http://azoresbioportal.uac.pt/azores-species/longiunguis-luzulella-8613/

##### Ecological interactions

###### Native status

Introduced

##### Distribution

SJG*

##### Notes

Biogeographical Realm: Western Palearctic

#### Myzus cerasi

(Fabricius, 1775)

http://azoresbioportal.uac.pt/azores-species/myzus-cerasi-8641/

##### Ecological interactions

###### Native status

Introduced

##### Distribution

FLO*; TER

##### Notes

Also present: MAD; CAN (Biogeographical Realm: Australian; Eastern Palearctic; Near East; Nearctic; North Africa; Oriental)

#### Neomyzus circumflexus

(Buckton, 1876)

http://azoresbioportal.uac.pt/azores-species/neomyzus-circumflexus-8644/

##### Ecological interactions

###### Native status

Introduced

##### Distribution

FLO*; TER; SMG

##### Notes

Also present: MAD (Biogeographical Realm: Cosmopolitan)

#### Pseudacaudella rubida

(Börner, 1939)

http://azoresbioportal.uac.pt/azores-species/pseudacaudella-rubida-8653/

##### Ecological interactions

###### Native status

Native

##### Distribution

FLO*; PIC; TER; SMG*; SMR

##### Notes

Biogeographical Realm: Nearctic

#### Rhopalosiphoninus latysiphon

(Davidson, 1912)

http://azoresbioportal.uac.pt/azores-species/rhopalosiphoninus-latysiphon-7155/

##### Ecological interactions

###### Native status

Introduced

##### Distribution

FLO*; FAI*; PIC*; GRA; SJG*; TER*; SMG; SMR*

##### Notes

Also present: MAD; CAN (Biogeographical Realm: Cosmopolitan)

#### Rhopalosiphum oxyacanthae

(Schrank, 1801)

http://azoresbioportal.uac.pt/azores-species/rhopalosiphum-oxyacanthae-8659/

##### Ecological interactions

###### Native status

Introduced

##### Distribution

FLO; FAI; PIC; GRA; SJG*; TER; SMG; SMR

##### Notes

Also present: MAD (Biogeographical Realm: Western Palaeartic; Japan)

#### Rhopalosiphum padi

(Linnaeus, 1758)

http://azoresbioportal.uac.pt/azores-species/rhopalosiphum-padi-8662/

##### Ecological interactions

###### Native status

Introduced

##### Distribution

COR; FLO; PIC; GRA; SJG; TER; SMG; SMR

##### Notes

Also present: MAD; CAN (Biogeographical Realm: Cosmopolitan)

#### Rhopalosiphum rufiabdominalis

(Sasaki, 1899)

http://azoresbioportal.uac.pt/azores-species/rhopalosiphum-rufiabdominale-8663/

##### Ecological interactions

###### Native status

Introduced

##### Distribution

COR; FLO; FAI; PIC*; GRA; SJG; TER*; SMG; SMR

##### Notes

Also present: MAD (Biogeographical Realm: Cosmopolitan)

#### Toxoptera aurantii

(Boyer de Fonscolombe, 1841)

http://azoresbioportal.uac.pt/azores-species/toxoptera-aurantii-8672/

##### Ecological interactions

###### Native status

Introduced

##### Distribution

FLO; FAI; PIC*; GRA; SJG; TER; SMG; SMR

##### Notes

Also present: MAD; CAN; CVP (Biogeographical Realm: Cosmopolitan)

#### Uroleucon erigeronense

(Thomas, 1878)

http://azoresbioportal.uac.pt/azores-species/uroleucon-erigeronense-8673/

##### Ecological interactions

###### Native status

Introduced

##### Distribution

SJG*; TER*; SMG*; SMR*

##### Notes

Biogeographical Realm: Eastern Palearctic; Near East; Nearctic; Neotropical; North Africa; Oriental

#### Cercopidae

#### Philaenus spumarius

(Linnaeus, 1758)

http://azoresbioportal.uac.pt/azores-species/philaenus-spumarius-8400/

##### Ecological interactions

###### Native status

Introduced

##### Distribution

TER; SMG

##### Notes

Also present: CAN (Biogeographical Realm: Cosmopolitan)

#### Cicadellidae

#### Anoscopus albifrons

(Linnaeus, 1758)

http://azoresbioportal.uac.pt/azores-species/anoscopus-albifrons-8403/

##### Ecological interactions

###### Native status

Native

##### Distribution

FLO*; FAI*; PIC; GRA; SJG*; TER; SMG; SMR

##### Notes

Also present: MAD; CAN (Biogeographical Realm: Holarctic)

#### Aphrodes hamiltoni

Quartau & Borges, 2003

http://azoresbioportal.uac.pt/azores-species/aphrodes-hamiltoni-8404/

##### Ecological interactions

###### Native status

Azores endemic

##### Distribution

FLO*; FAI*; PIC*; GRA; SJG*; TER*; SMG*; SMR*

##### Notes

Biogeographical Realm: Western Palearctic (Macaronesia)

#### Eupteryx azorica

Ribaut, 1941

http://azoresbioportal.uac.pt/azores-species/eupteryx-azorica-6899/

##### Ecological interactions

###### Native status

Azores endemic

##### Distribution

COR; FLO; PIC; GRA; SJG; TER; SMG

##### Notes

Biogeographical Realm: Western Palearctic (Macaronesia)

#### Opsius stactogallus

Fieber, 1866

http://azoresbioportal.uac.pt/azores-species/opsius-stactogalus-8414/

##### Ecological interactions

###### Native status

Native

##### Distribution

COR; FLO*; FAI; PIC; GRA; SJG; TER; SMR

##### Notes

Also present: CAN (Biogeographical Realm: Holarctic)

#### Cixiidae

#### Cixius azofloresi

Remane & Asche, 1979

http://azoresbioportal.uac.pt/azores-species/cixius-azofloresi-8420/

##### Ecological interactions

###### Native status

Azores endemic

##### Distribution

COR; FLO

##### Notes

Biogeographical Realm: Western Palearctic (Macaronesia)

#### Cixius azomariae

Remane & Asche, 1979

http://azoresbioportal.uac.pt/azores-species/cixius-azomariae-8417/

##### Ecological interactions

###### Native status

Azores endemic

##### Distribution

SMR

##### Notes

Biogeographical Realm: Western Palearctic (Macaronesia)

#### Cixius azopifajo azofa

Remane & Asche, 1979

http://azoresbioportal.uac.pt/azores-species/cixius-azopifajo-azofa-13516/

##### Ecological interactions

###### Native status

Azores endemic

##### Distribution

FAI

##### Notes

Biogeographical Realm: Western Palearctic (Macaronesia)

#### Cixius azopifajo azojo

Remane & Asche, 1979

http://azoresbioportal.uac.pt/azores-species/cixius-azopifajo-azojo-13518/

##### Ecological interactions

###### Native status

Azores endemic

##### Distribution

SJG

##### Notes

Biogeographical Realm: Western Palearctic (Macaronesia)

#### Cixius azopifajo azopifajo

Remane & Asche, 1979

http://azoresbioportal.uac.pt/azores-species/cixius-azopifajo-azopifajo-13517/

##### Ecological interactions

###### Native status

Azores endemic

##### Distribution

PIC

##### Notes

Biogeographical Realm: Western Palearctic (Macaronesia)

#### Cixius azoricus azoricus

Lindberg, 1954

http://azoresbioportal.uac.pt/azores-species/cixius-azoricus-azoricus-13532/

##### Ecological interactions

###### Native status

Azores endemic

##### Distribution

FAI; SJG; TER; SMG*

##### Notes

Biogeographical Realm: Western Palearctic (Macaronesia)

#### Cixius azoricus azoropicoi

Remane & Asche, 1979

http://azoresbioportal.uac.pt/azores-species/cixius-azoricus-azoropicoi-13519/

##### Ecological interactions

###### Native status

Azores endemic

##### Distribution

PIC

##### Notes

Biogeographical Realm: Western Palearctic (Macaronesia)

#### Cixius azoterceirae

Remane & Asche, 1979

http://azoresbioportal.uac.pt/azores-species/cixius-azoterceirae-7099/

##### Ecological interactions

###### Native status

Azores endemic

##### Distribution

TER

##### Notes

Biogeographical Realm: Western Palearctic (Macaronesia)

#### Cixius insularis

Lindberg, 1954

http://azoresbioportal.uac.pt/azores-species/cixius-insularis-8419/

##### Ecological interactions

###### Native status

Azores endemic

##### Distribution

SMG

##### Notes

Biogeographical Realm: Western Palearctic (Macaronesia)

#### Cydnidae

#### Geotomus punctulatus

(A. Costa, 1847)

http://azoresbioportal.uac.pt/azores-species/geotomus-punctulatus-8440/

##### Ecological interactions

###### Native status

Native

##### Distribution

FAI*; GRA; TER; SMG; SMR

##### Notes

Also present: MAD; CAN (Biogeographical Realm: Western Palearctic)

#### Delphacidae

#### Megamelodes quadrimaculatus

(Signoret, 1865)

http://azoresbioportal.uac.pt/azores-species/megamelodes-quadrimaculatus-8427/

##### Ecological interactions

###### Native status

Native

##### Distribution

FLO*; FAI*; PIC; GRA; SJG*; TER; SMG*; SMR

##### Notes

Also present: MAD (Biogeographical Realm: Western Palearctic (Macaronesia))

#### Drepanosiphidae

#### Anoecia corni

(Fabricius, 1775)

http://azoresbioportal.uac.pt/azores-species/anoecia-corni-8629/

##### Ecological interactions

###### Native status

Introduced

##### Distribution

FLO; PIC; SJG*; TER; SMG; SMR

##### Notes

Also present: MAD; CAN (Biogeographical Realm: Cosmopolitan (except Australia))

#### Flatidae

#### Cyphopterum adcendens

(Herrich-Schaeffer, 1835)

http://azoresbioportal.uac.pt/azores-species/cyphopterum-adcendens-7089/

##### Ecological interactions

###### Native status

Native

##### Distribution

COR; FLO; FAI; PIC; GRA; SJG; TER; SMG; SMR*

##### Notes

Biogeographical Realm: Western Palearctic

#### Lachnidae

#### Cinara juniperi

(De Geer, 1773)

http://azoresbioportal.uac.pt/azores-species/cinara-juniperi-7078/

##### Ecological interactions

###### Native status

Native

##### Distribution

COR; FLO*; FAI*; PIC; SJG*; TER; SMG; SMR

##### Notes

Also present: MAD (Biogeographical Realm: Holarctic)

#### Lygaeidae

#### Beosus maritimus

(Scopoli, 1763)

http://azoresbioportal.uac.pt/azores-species/beosus-maritimus-8446/

##### Ecological interactions

###### Native status

Native

##### Distribution

FLO; FAI; TER; SMR

##### Notes

Also present: MAD; CAN (Biogeographical Realm: Western Palearctic)

#### Gastrodes grossipes grossipes

(De Geer, 1773)

http://azoresbioportal.uac.pt/azores-species/gastrodes-grossipes-grossipes-13559/

##### Ecological interactions

###### Native status

Introduced

##### Distribution

TER*

##### Notes

Biogeographical Realm: Palearctic

#### Heterogaster urticae

(Fabricius, 1775)

http://azoresbioportal.uac.pt/azores-species/heterogaster-urticae-8449/

##### Ecological interactions

###### Native status

Native

##### Distribution

PIC; TER*; SMG

##### Notes

Also present: MAD (Biogeographical Realm: Western Palearctic)

#### Kleidocerys ericae

(Horváth, 1908)

http://azoresbioportal.uac.pt/azores-species/kleidocerys-ericae-7157/

##### Ecological interactions

###### Native status

Native

##### Distribution

COR; FLO; FAI; PIC; GRA; SJG*; TER; SMG; SMR*

##### Notes

Also present: MAD; CAN (Biogeographical Realm: Western Palearctic)

#### Microplax plagiata

(Fieber, 1837)

http://azoresbioportal.uac.pt/azores-species/microplax-plagiata-8451/

##### Ecological interactions

###### Native status

Native

##### Distribution

SMR*

##### Notes

Also present: CAN (Biogeographical Realm: Palearctic)

#### Nysius atlantidum

Horváth, 1990

http://azoresbioportal.uac.pt/azores-species/nysius-atlantidum-7085/

##### Ecological interactions

###### Native status

Azores endemic

##### Distribution

FLO; FAI; GRA; TER; SMG; SMR*

##### Notes

Biogeographical Realm: Western Palearctic (Macaronesia)

#### Nysius ericae ericae

Schilling, 1829

http://azoresbioportal.uac.pt/azores-species/nysius-ericae-ericae-13521/

##### Ecological interactions

###### Native status

Native

##### Distribution

COR; FLO; FAI; PIC; GRA; SJG; TER; SMG; SMR

##### Notes

Also present: MAD; CAN (Biogeographical Realm: Afro-tropical)

#### Plinthisus brevipennis

(Latreille, 1807)

http://azoresbioportal.uac.pt/azores-species/plinthisus-brevipennis-8452/

##### Ecological interactions

###### Native status

Native

##### Distribution

FAI*; PIC; GRA; SMG; SMR

##### Notes

Also present: MAD (Biogeographical Realm: Western Palearctic)

#### Plinthisus minutissimus

Fieber, 1864

http://azoresbioportal.uac.pt/azores-species/plinthisus-minutissimus-7151/

##### Ecological interactions

###### Native status

Native

##### Distribution

FAI*; TER

##### Notes

Biogeographical Realm: Western Palearctic

#### Scolopostethus decoratus

(Hahn, 1833)

http://azoresbioportal.uac.pt/azores-species/scolopostethus-decoratus-7097/

##### Ecological interactions

###### Native status

Native

##### Distribution

FLO; FAI*; PIC; GRA; TER; SMG; SMR

##### Notes

Biogeographical Realm: Palearctic

#### Microphysidae

#### Loricula coleoptrata

(Fallén, 1807)

http://azoresbioportal.uac.pt/azores-species/loricula-coleoptrata-8458/

##### Ecological interactions

###### Native status

Native

##### Distribution

FAI; SMG*; SMR

##### Notes

Biogeographical Realm: Western Palearctic

#### Loricula elegantula

(Bärensprung, 1858)

http://azoresbioportal.uac.pt/azores-species/loricula-elegantula-8445/

##### Ecological interactions

###### Native status

Native

##### Distribution

FLO; PIC*; GRA; SMG*; SMR

##### Notes

Biogeographical Realm: Western Palearctic

#### Miridae

#### Campyloneura virgula

(Herrich-Schaeffer, 1835)

http://azoresbioportal.uac.pt/azores-species/campyloneura-virgula-8460/

##### Ecological interactions

###### Native status

Native

##### Distribution

FLO; FAI; PIC; GRA; SJG*; TER; SMG

##### Notes

Biogeographical Realm: Nearctic

#### Closterotomus norwegicus

(Gmelin, 1790)

http://azoresbioportal.uac.pt/azores-species/closterotomus-norwegicus-8461/

##### Ecological interactions

###### Native status

Native

##### Distribution

FLO; FAI; PIC; TER; SMR*

##### Notes

Also present: MAD; CAN (Biogeographical Realm: Australian; Nearctic)

#### Heterotoma planicornis

(Pallas, 1772)

http://azoresbioportal.uac.pt/azores-species/heterotoma-planicornis-8462/

##### Ecological interactions

###### Native status

Native

##### Distribution

FAI; PIC; GRA; TER; SMG; SMR

##### Notes

Biogeographical Realm: Nearctic

#### Monalocoris filicis

(Linnaeus, 1758)

http://azoresbioportal.uac.pt/azores-species/monalocoris-filicis-8465/

##### Ecological interactions

###### Native status

Native

##### Distribution

COR; FLO; FAI; PIC; GRA; SJG; TER; SMG; SMR

##### Notes

Biogeographical Realm: Holactic; Afro-tropical; Northern Asia (except China

#### Pinalitus oromii

J. Ribes, 1992

http://azoresbioportal.uac.pt/azores-species/pinalitus-oromii-7093/

##### Ecological interactions

###### Native status

Azores endemic

##### Distribution

FLO*; FAI; PIC; GRA; SJG; TER*; SMG; SMR*

##### Notes

Biogeographical Realm: Western Palearctic (Macaronesia)

#### Polymerus cognatus

(Fieber, 1858)

http://azoresbioportal.uac.pt/azores-species/polymerus-cognatus-8474/

##### Ecological interactions

###### Native status

Native

##### Distribution

COR; FLO*; FAI; PIC; GRA; SJG; TER; SMG; SMR

##### Notes

Biogeographical Realm: Nearctic

#### Polymerus vulneratus

(Panzer, 1806)

http://azoresbioportal.uac.pt/azores-species/polymerus-vulneratus-8475/

##### Ecological interactions

###### Native status

Native

##### Distribution

PIC*; TER

##### Notes

Biogeographical Realm: Nearctic

#### Nabidae

#### Nabis pseudoferus ibericus

Remane, 1962

http://azoresbioportal.uac.pt/azores-species/nabis-pseudoferus-ibericus-13443/

##### Ecological interactions

###### Native status

Native

##### Distribution

COR; FLO; FAI; PIC; GRA; SJG; TER; SMG; SMR

##### Notes

Also present: MAD; CAN (Biogeographical Realm: Western Palearctic)

#### Pentatomidae

#### Nezara viridula

(Linnaeus, 1758)

http://azoresbioportal.uac.pt/azores-species/nezara-viridula-8482/

##### Ecological interactions

###### Native status

Introduced

##### Distribution

COR; FLO; FAI; PIC; GRA; SJG; TER; SMG; SMR

##### Notes

Also present: MAD; CAN; CVP (Biogeographical Realm: Cosmopolitan)

#### Psyllidae

#### Acizzia uncatoides

(Ferris & Klyver, 1932)

http://azoresbioportal.uac.pt/azores-species/acizzia-uncatoides-8547/

##### Ecological interactions

###### Native status

Introduced

##### Distribution

PIC; GRA; TER*

##### Notes

Also present: CAN (Biogeographical Realm: Australian)

#### Cacopsylla pulchella

(Löw, 1877)

http://azoresbioportal.uac.pt/azores-species/cacopsylla-pulchella-8527/

##### Ecological interactions

###### Native status

Introduced

##### Distribution

PIC*

##### Notes

Biogeographical Realm: Western Palearctic

#### Strophingia harteni

Hodkinson, 1981

http://azoresbioportal.uac.pt/azores-species/strophingia-harteni-7087/

##### Ecological interactions

###### Native status

Azores endemic

##### Distribution

COR; FLO; FAI*; PIC*; GRA; SJG*; TER*; SMG; SMR*

##### Notes

Biogeographical Realm: Western Palearctic (Macaronesia)

#### Reduviidae

#### Empicoris rubromaculatus

(Blackburn, 1889)

http://azoresbioportal.uac.pt/azores-species/empicoris-rubromaculatus-8483/

##### Ecological interactions

###### Native status

Introduced

##### Distribution

PIC; TER*; SMG; SMR

##### Notes

Also present: MAD; CAN (Biogeographical Realm: Afro-tropical; Australian; Neotropical; Oriental)

#### Saldidae

#### Saldula palustris

(Douglas, 1874)

http://azoresbioportal.uac.pt/azores-species/saldula-palustris-8471/

##### Ecological interactions

###### Native status

Native

##### Distribution

TER; SMG

##### Notes

Also present: MAD; CAN (Biogeographical Realm: Afro-tropical; Eastern Palearctic; Near East; North Africa)

#### Tingidae

#### Acalypta parvula

(Fallén, 1807)

http://azoresbioportal.uac.pt/azores-species/acalypta-parvula-8491/

##### Ecological interactions

###### Native status

Native

##### Distribution

FLO; FAI; PIC; TER; SMG

##### Notes

Also present: MAD; CAN (Biogeographical Realm: Nearctic; North Africa (except Sinai Peninsula))

#### Triozidae

#### Trioza laurisilvae

Hodkinson, 1990

http://azoresbioportal.uac.pt/azores-species/trioza-laurisilvae-7090/

##### Ecological interactions

###### Native status

Native

##### Distribution

FLO*; FAI*; PIC; GRA; SJG*; TER*; SMG; SMR*

##### Notes

Also present: MAD; CAN (Biogeographical Realm: Western Palearctic (Macaronesia))

#### Thysanoptera

#### Aeolothripidae

#### Aeolothrips collaris

Priesner, 1919

http://azoresbioportal.uac.pt/azores-species/aeolothrips-collaris-8269/

##### Ecological interactions

###### Native status

Native

##### Distribution

FAI; PIC; SJG; TER; SMG; SMR

##### Notes

Also present: MAD; CAN (Biogeographical Realm: Palearctic)

#### Aeolothrips gloriosus

Bagnall, 1914

http://azoresbioportal.uac.pt/azores-species/aeolothrips-gloriosus-8271/

##### Ecological interactions

###### Native status

Native

##### Distribution

FAI; PIC; GRA; SJG; TER; SMG; SMR

##### Notes

Biogeographical Realm: Palearctic

#### Phlaeothripidae

#### Eurythrips tristis

Hood, 1941

http://azoresbioportal.uac.pt/azores-species/eurythrips-tristis-8370/

##### Ecological interactions

###### Native status

Introduced

##### Distribution

SJG*; TER*

##### Notes

Biogeographical Realm: Nearctic

#### Hoplandrothrips consobrinus

(Knechtel, 1951)

http://azoresbioportal.uac.pt/azores-species/hoplandrothrips-consobrinus-8371/

##### Ecological interactions

###### Native status

Introduced

##### Distribution

SJG*; TER; SMG

##### Notes

Also present: MAD; CAN (Biogeographical Realm: Palearctic)

#### Hoplothrips corticis

(De Geer, 1773)

http://azoresbioportal.uac.pt/azores-species/hoplothrips-corticis-7086/

##### Ecological interactions

###### Native status

Native

##### Distribution

FLO; FAI; PIC; GRA; SJG; TER; SMG; SMR

##### Notes

Biogeographical Realm: Cosmopolitan

#### Hoplothrips ulmi

(Fabricius, 1781)

http://azoresbioportal.uac.pt/azores-species/hoplothrips-ulmi-8382/

##### Ecological interactions

###### Native status

Introduced

##### Distribution

FLO*; FAI; SJG*; TER; SMG*

##### Notes

Also present: MAD (Biogeographical Realm: Palearctic)

#### Nesothrips propinquus

(Bagnall, 1916)

http://azoresbioportal.uac.pt/azores-species/nesothrips-propinquus-8383/

##### Ecological interactions

###### Native status

Introduced

##### Distribution

FAI; PIC; SJG; TER; SMG; SMR

##### Notes

Also present: MAD; CAN (Biogeographical Realm: Cosmopolitan)

#### Thripidae

#### Aptinothrips rufus

Haliday, 1836

http://azoresbioportal.uac.pt/azores-species/aptinothrips-rufus-8365/

##### Ecological interactions

###### Native status

Introduced

##### Distribution

FLO; FAI; PIC; GRA; SJG; TER; SMG; SMR

##### Notes

Also present: MAD; CAN (Biogeographical Realm: Cosmopolitan)

#### Ceratothrips ericae

(Haliday, 1836)

http://azoresbioportal.uac.pt/azores-species/ceratothrips-ericae-8366/

##### Ecological interactions

###### Native status

Native

##### Distribution

FAI; PIC; SJG; TER; SMG; SMR

##### Notes

Also present: MAD (Biogeographical Realm: Holarctic)

#### Heliothrips haemorrhoidalis

(Bouché, 1833)

http://azoresbioportal.uac.pt/azores-species/heliothrips-haemorrhoidalis-7080/

##### Ecological interactions

###### Native status

Introduced

##### Distribution

FLO; FAI; PIC; GRA; SJG; TER; SMG; SMR

##### Notes

Also present: MAD; CAN; CVP (Biogeographical Realm: Cosmopolitan)

#### Hercinothrips bicinctus

(Bagnall, 1919)

http://azoresbioportal.uac.pt/azores-species/hercinothrips-bicinctus-7092/

##### Ecological interactions

###### Native status

Introduced

##### Distribution

FAI; PIC; GRA; SJG; TER; SMG; SMR

##### Notes

Also present: MAD; CAN (Biogeographical Realm: Cosmopolitan)

#### Isoneurothrips australis

Bagnall, 1915

http://azoresbioportal.uac.pt/azores-species/isoneurothrips-australis-8387/

##### Ecological interactions

###### Native status

Introduced

##### Distribution

TER; SMG; SMR

##### Notes

Also present: MAD; CAN (Biogeographical Realm: Cosmopolitan)

#### Thrips atratus

Haliday, 1836

http://azoresbioportal.uac.pt/azores-species/thrips-atratus-8392/

##### Ecological interactions

###### Native status

Native

##### Distribution

FAI; PIC; SJG; TER; SMG; SMR

##### Notes

Also present: MAD (Biogeographical Realm: Holarctic)

#### Thrips flavus

Schrank, 1776

http://azoresbioportal.uac.pt/azores-species/thrips-flavus-8394/

##### Ecological interactions

###### Native status

Native

##### Distribution

FAI; PIC; SJG; TER; SMG; SMR

##### Notes

Also present: MAD (Biogeographical Realm: Holarctic)

#### Neuroptera

#### Hemerobiidae

#### Hemerobius azoricus

Tjeder, 1948

http://azoresbioportal.uac.pt/azores-species/hemerobius-azoricus-6862/

##### Ecological interactions

###### Native status

Azores endemic

##### Distribution

FLO*; FAI*; PIC; GRA; SJG; TER*; SMG; SMR

##### Notes

Biogeographical Realm: Western Palearctic (Macaronesia)

#### Coleoptera

#### Anobiidae

#### Anobium punctatum

(De Gueer, 1774)

http://azoresbioportal.uac.pt/azores-species/anobium-punctatum-7571/

##### Ecological interactions

###### Native status

Introduced

##### Distribution

COR; FLO; FAI; PIC; GRA; TER; SMG; SMR

##### Notes

Also present: MAD; CAN (Biogeographical Realm: Western Palearctic)

#### Brentidae

#### Aspidapion radiolus chalybeipenne

(Wollaston, 1854)

http://azoresbioportal.uac.pt/azores-species/aspidapion-radiolus-chalybeipenne-13444/

##### Ecological interactions

###### Native status

Native

##### Distribution

COR; FLO; FAI; SJG; TER; SMG; SMR

##### Notes

Also present: MAD; CAN (Biogeographical Realm: Palearctic)

#### Carabidae

#### Acupalpus dubius

Schilsky, 1888

http://azoresbioportal.uac.pt/azores-species/acupalpus-dubius-7371/

##### Ecological interactions

###### Native status

Native

##### Distribution

FLO; FAI; GRA; SJG*; TER; SMG; SMR

##### Notes

Also present: MAD (Biogeographical Realm: Western Palearctic)

#### Acupalpus flavicollis

(Sturm, 1825)

http://azoresbioportal.uac.pt/azores-species/acupalpus-flavicollis-7306/

##### Ecological interactions

###### Native status

Native

##### Distribution

FAI*; TER

##### Notes

Biogeographical Realm: Palearctic

#### Amara aenea

(De Geer, 1774)

http://azoresbioportal.uac.pt/azores-species/amara-aenea-7366/

##### Ecological interactions

###### Native status

Introduced

##### Distribution

COR; FLO; FAI; PIC; GRA; SJG; TER; SMG; SMR

##### Notes

Also present: MAD; CAN; CVP (Biogeographical Realm: Holarctic)

#### Anisodactylus binotatus

(Fabricius, 1787)

http://azoresbioportal.uac.pt/azores-species/anisodactylus-binotatus-7367/

##### Ecological interactions

###### Native status

Introduced

##### Distribution

COR; FLO; FAI; PIC; GRA; SJG; TER; SMG; SMR

##### Notes

Also present: MAD (Biogeographical Realm: Holarctic)

#### Calathus lundbladi

Colas, 1938

http://azoresbioportal.uac.pt/azores-species/calathus-lundbladi-7354/

##### Ecological interactions

###### Native status

Azores endemic

##### Distribution

SMG

##### Notes

Biogeographical Realm: Western Palearctic (Macaronesia)

#### Cedrorum azoricus azoricus

Borges & Serrano, 1993

http://azoresbioportal.uac.pt/azores-species/cedrorum-azoricus-azoricus-13442/

##### Ecological interactions

###### Native status

Azores endemic

##### Distribution

TER; SMR

##### Notes

Biogeographical Realm: Western Palearctic (Macaronesia)

#### Cedrorum azoricus caveirensis

Borges & Serrano, 1993

http://azoresbioportal.uac.pt/azores-species/cedrorum-azoricus-caveirensis-13453/

##### Ecological interactions

###### Native status

Azores endemic

##### Distribution

PIC

##### Notes

Biogeographical Realm: Western Palearctic (Macaronesia)

#### Laemostenus complanatus

(Dejean, 1828)

http://azoresbioportal.uac.pt/azores-species/laemostenus-complanatus-7362/

##### Ecological interactions

###### Native status

Introduced

##### Distribution

FLO; FAI; PIC; GRA; SJG; TER; SMG; SMR

##### Notes

Also present: MAD; CAN (Biogeographical Realm: Cosmopolitan)

#### Ocys harpaloides

(Audinet-Serville, 1821)

http://azoresbioportal.uac.pt/azores-species/ocys-harpaloides-7335/

##### Ecological interactions

###### Native status

Native

##### Distribution

COR; FLO; FAI; PIC; GRA; SJG; TER; SMG; SMR

##### Notes

Also present: MAD (Biogeographical Realm: Western Palearctic)

#### Paranchus albipes

(Fabricius, 1796)

http://azoresbioportal.uac.pt/azores-species/paranchus-albipes-7350/

##### Ecological interactions

###### Native status

Introduced

##### Distribution

COR; FLO; FAI; PIC; GRA; SJG; TER; SMG; SMR

##### Notes

Also present: MAD; CAN (Biogeographical Realm: Holarctic)

#### Pseudanchomenus aptinoides

Tarnier, 1860

http://azoresbioportal.uac.pt/azores-species/pseudanchomenus-aptinoides-6831/

##### Ecological interactions

###### Native status

Azores endemic

##### Distribution

PIC; SMG

##### Notes

Biogeographical Realm: Western Palearctic (Macaronesia)

#### Pseudoophonus rufipes

De Geer, 1774

http://azoresbioportal.uac.pt/azores-species/pseudoophonus-rufipes-7373/

##### Ecological interactions

###### Native status

Introduced

##### Distribution

COR; FLO; FAI; PIC; GRA; SJG; TER; SMG; SMR

##### Notes

Also present: MAD (Biogeographical Realm: Western Palearctic)

#### Pterostichus aterrimus aterrimus

(Herbst, 1784)

http://azoresbioportal.uac.pt/azores-species/pterostichus-aterrimus-aterrimus-13452/

##### Ecological interactions

###### Native status

Native

##### Distribution

PIC*; SJG; TER

##### Notes

Also present: MAD (Biogeographical Realm: Palearctic)

#### Pterostichus vernalis

(Panzer, 1796)

http://azoresbioportal.uac.pt/azores-species/pterostichus-vernalis-7348/

##### Ecological interactions

###### Native status

Introduced

##### Distribution

FAI; PIC; GRA; SJG; TER; SMG

##### Notes

Biogeographical Realm: Palearctic

#### Stenolophus teutonus

(Schrank, 1781)

http://azoresbioportal.uac.pt/azores-species/stenolophus-teutonus-7368/

##### Ecological interactions

###### Native status

Native

##### Distribution

COR; FLO; FAI; GRA; SJG; TER; SMG; SMR

##### Notes

Also present: MAD; CAN (Biogeographical Realm: Western Palearctic)

#### Trechus terrabravensis

Borges, Serrano & Amorim, 2004

http://azoresbioportal.uac.pt/azores-species/trechus-terrabravensis-7345/

##### Ecological interactions

###### Native status

Azores endemic

##### Distribution

TER

##### Notes

Biogeographical Realm: Western Palearctic (Macaronesia)

#### Cerambycidae

#### Crotchiella brachyptera

Israelson, 1985

http://azoresbioportal.uac.pt/azores-species/crotchiella-brachyptera-7879/

##### Ecological interactions

###### Native status

Azores endemic

##### Distribution

PIC; TER; SMG; SMR

##### Notes

Biogeographical Realm: Western Palearctic (Macaronesia)

#### Chrysomelidae

#### Chaetocnema hortensis

(Fourcroy , 1785)

http://azoresbioportal.uac.pt/azores-species/chaetocnema-hortensis-7888/

##### Ecological interactions

###### Native status

Introduced

##### Distribution

FLO; FAI; PIC; GRA; SJG; TER; SMG; SMR

##### Notes

Also present: MAD (Biogeographical Realm: Western Palearctic)

#### Epitrix hirtipennis

(Melsheimer, 1847)

http://azoresbioportal.uac.pt/azores-species/epitrix-hirtipennis-7886/

##### Ecological interactions

###### Native status

Introduced

##### Distribution

PIC; GRA; TER; SMG; SMR

##### Notes

Biogeographical Realm: Nearctic

#### Psylliodes marcidus

(Illiger, 1807)

http://azoresbioportal.uac.pt/azores-species/psylliodes-marcidus-7916/

##### Ecological interactions

###### Native status

Native

##### Distribution

FLO; FAI; PIC; GRA; TER; SMG; SMR

##### Notes

Also present: MAD (Biogeographical Realm: Palearctic)

#### Ciidae

#### Atlantocis gillerforsi

Israelson, 1986

http://azoresbioportal.uac.pt/azores-species/atlantocis-gillerforsi-7674/

##### Ecological interactions

###### Native status

Azores endemic

##### Distribution

FLO; PIC; TER*; SMG; SMR

##### Notes

Biogeographical Realm: Western Palearctic (Macaronesia)

#### Coccinellidae

#### Clitostethus arcuatus

(Rossi, 1794)

http://azoresbioportal.uac.pt/azores-species/clitostethus-arcuatus-7638/

##### Ecological interactions

###### Native status

Introduced

##### Distribution

GRA; SJG; TER; SMG; SMR

##### Notes

Also present: MAD; CAN (Biogeographical Realm: Western Palearctic)

#### Coccinella undecimpunctata undecimpunctata

Linnaeus, 1758

http://azoresbioportal.uac.pt/azores-species/coccinella-undecimpunctata-undecimpunctata-13471/

##### Ecological interactions

###### Native status

Introduced

##### Distribution

COR; FLO; FAI; PIC; GRA; SJG; TER; SMG; SMR

##### Notes

Biogeographical Realm: Holarctic

#### Lindorus lophanthae

(Blaisdell, 1892)

http://azoresbioportal.uac.pt/azores-species/lindorus-lophanthae-7649/

##### Ecological interactions

###### Native status

Introduced

##### Distribution

FLO; GRA; SJG*; TER; SMG; SMR

##### Notes

Also present: MAD; CAN (Biogeographical Realm: Cosmopolitan)

#### Rodolia cardinalis

(Mulsant, 1850)

http://azoresbioportal.uac.pt/azores-species/rodolia-cardinalis-7648/

##### Ecological interactions

###### Native status

Introduced

##### Distribution

COR; FLO; PIC; GRA; SJG; TER; SMG; SMR

##### Notes

Also present: MAD; CAN; CVP (Biogeographical Realm: Cosmopolitan)

#### Corylophidae

#### Sericoderus lateralis

(Gyllenhal, 1827)

http://azoresbioportal.uac.pt/azores-species/sericoderus-lateralis-7672/

##### Ecological interactions

###### Native status

Introduced

##### Distribution

COR; FLO; FAI; PIC; GRA; SJG; TER; SMG; SMR

##### Notes

Also present: MAD; CAN; CVP (Biogeographical Realm: Palearctic)

#### Curculionidae

#### Calacalles subcarinatus

(Israelson, 1984)

http://azoresbioportal.uac.pt/azores-species/calacalles-subcarinatus-6896/

##### Ecological interactions

###### Native status

Azores endemic

##### Distribution

COR; FLO; FAI*; PIC; GRA; SJG*; TER; SMG; SMR

##### Notes

Biogeographical Realm: Western Palearctic (Macaronesia)

#### Caulotrupis parvus

Israelson, 1985

http://azoresbioportal.uac.pt/azores-species/caulotrupis-parvus-7942/

##### Ecological interactions

###### Native status

Azores endemic

##### Distribution

SMR

##### Notes

Biogeographical Realm: Western Palearctic (Macaronesia)

#### Coccotrypes carpophagus

(Hornung, 1842)

http://azoresbioportal.uac.pt/azores-species/coccotrypes-carpophagus-7872/

##### Ecological interactions

###### Native status

Introduced

##### Distribution

FAI; PIC*; GRA; TER; SMG; SMR

##### Notes

Also present: MAD; CAN (Biogeographical Realm: Cosmopolitan)

#### Drouetius borgesi borgesi

Machado, 2009

http://azoresbioportal.uac.pt/azores-species/drouetius-borgesi-borgesi-13569/

##### Ecological interactions

###### Native status

Azores endemic

##### Distribution

TER

##### Notes

Biogeographical Realm: Western Palearctic (Macaronesia)

#### Drouetius borgesi centralis

Machado, 2009

http://azoresbioportal.uac.pt/azores-species/drouetius-borgesi-centralis-13568/

##### Ecological interactions

###### Native status

Azores endemic

##### Distribution

FAI; PIC; GRA; SJG

##### Notes

Biogeographical Realm: Western Palearctic (Macaronesia)

#### Drouetius borgesi sanctmichaelis

Machado, 2009

http://azoresbioportal.uac.pt/azores-species/drouetius-borgesi-sanctmichaelis-13566/

##### Ecological interactions

###### Native status

Azores endemic

##### Distribution

SMG

##### Notes

Biogeographical Realm: Western Palearctic (Macaronesia)

#### Gymnetron pascuorum

(Gyllenhal, 1813)

http://azoresbioportal.uac.pt/azores-species/gymnetron-pascuorum-7951/

##### Ecological interactions

###### Native status

Introduced

##### Distribution

FAI; TER; SMR

##### Notes

Also present: MAD; CAN (Biogeographical Realm: Western Palearctic)

#### Orthochaetes insignis

(Aubé, 1863)

http://azoresbioportal.uac.pt/azores-species/orthochaetes-insignis-7946/

##### Ecological interactions

###### Native status

Native

##### Distribution

FLO; FAI*; TER; SMR

##### Notes

Biogeographical Realm: Palearctic

#### Otiorhynchus cribricollis

Gyllenhal, 1834

http://azoresbioportal.uac.pt/azores-species/otiorhynchus-cribricollis-7907/

##### Ecological interactions

###### Native status

Introduced

##### Distribution

COR; FLO; FAI; PIC; GRA; SJG; TER; SMG; SMR

##### Notes

Also present: MAD; CAN (Biogeographical Realm: Western Palearctic)

#### Otiorhynchus rugosostriatus

(Goeze, 1777)

http://azoresbioportal.uac.pt/azores-species/otiorhynchus-rugosostriatus-7912/

##### Ecological interactions

###### Native status

Introduced

##### Distribution

FLO*; FAI; PIC; SJG*; TER; SMG; SMR

##### Notes

Also present: MAD (Biogeographical Realm: Palearctic)

#### Phloeosinus gillerforsi

Bright, 1987

http://azoresbioportal.uac.pt/azores-species/phloeosinus-gillerforsi-7163/

##### Ecological interactions

###### Native status

Azores endemic

##### Distribution

FLO; PIC; SJG*; TER*; SMG

##### Notes

Also present: CAN (Biogeographical Realm: Western Palearctic (Macaronesia))

#### Pseudechinosoma nodosum

Hustache, 1936

http://azoresbioportal.uac.pt/azores-species/pseudechinosoma-nodosum-6827/

##### Ecological interactions

###### Native status

Azores endemic

##### Distribution

FLO; FAI*; PIC; SJG*; TER*; SMG; SMR

##### Notes

Biogeographical Realm: Western Palearctic (Macaronesia)

#### Pseudophloeophagus aenopiceus

(Boheman, 1845)

http://azoresbioportal.uac.pt/azores-species/pseudophloeophagus-aenopiceus-7949/

##### Ecological interactions

###### Native status

Native

##### Distribution

FLO; FAI; PIC; GRA; SJG; TER; SMG; SMR

##### Notes

Also present: MAD (Biogeographical Realm: Palearctic)

#### Pseudophloeophagus tenax

Wollaston, 1854

http://azoresbioportal.uac.pt/azores-species/pseudophloeophagus-tenax-7094/

##### Ecological interactions

###### Native status

Native

##### Distribution

COR; FLO; FAI; PIC; GRA; SJG; TER; SMG; SMR

##### Notes

Also present: MAD (Biogeographical Realm: Western Palearctic (Macaronesia))

#### Sitona discoideus

Gyllenhal, 1834

http://azoresbioportal.uac.pt/azores-species/sitona-discoideus-7925/

##### Ecological interactions

###### Native status

Introduced

##### Distribution

FLO; FAI; GRA; SJG; TER*; SMG; SMR

##### Notes

Also present: MAD; CAN (Biogeographical Realm: Palearctic)

#### Xyleborinus alni

Nijima, 1909

http://azoresbioportal.uac.pt/azores-species/xyleborinus-alni-7920/

##### Ecological interactions

###### Native status

Introduced

##### Distribution

FLO; FAI; PIC; GRA; SJG; TER; SMG; SMR

##### Notes

Also present: MAD (Biogeographical Realm: Holarctic)

#### Dryophthoridae

#### Sitophilus oryzae

(Linnaeus, 1763)

http://azoresbioportal.uac.pt/azores-species/sitophilus-oryzae-7910/

##### Ecological interactions

###### Native status

Introduced

##### Distribution

FLO; FAI; PIC; GRA; SJG; TER; SMG; SMR

##### Notes

Also present: MAD; CAN; CVP (Biogeographical Realm: Cosmopolitan)

#### Dryopidae

#### Dryops algiricus

(Lucas, 1846)

http://azoresbioportal.uac.pt/azores-species/dryops-algiricus-7545/

##### Ecological interactions

###### Native status

Native

##### Distribution

FLO; TER; SMG; SMR

##### Notes

Biogeographical Realm: Palearctic

#### Dryops luridus

(Erichson, 1847)

http://azoresbioportal.uac.pt/azores-species/dryops-luridus-7546/

##### Ecological interactions

###### Native status

Native

##### Distribution

COR; FLO; FAI*; GRA; TER*; SMG; SMR

##### Notes

Also present: MAD; CAN (Biogeographical Realm: Palearctic)

#### Dytiscidae

#### Agabus bipustulatus

(Linnaeus, 1767)

http://azoresbioportal.uac.pt/azores-species/agabus-bipustulatus-7393/

##### Ecological interactions

###### Native status

Native

##### Distribution

FLO; PIC; SJG; TER

##### Notes

Also present: MAD (Biogeographical Realm: Palearctic)

#### Agabus godmani

Crotch, 1867

http://azoresbioportal.uac.pt/azores-species/agabus-godmani-7395/

##### Ecological interactions

###### Native status

Azores endemic

##### Distribution

FLO; FAI; PIC; GRA; SJG; TER; SMG

##### Notes

Biogeographical Realm: Western Palearctic (Macaronesia)

#### Hydroporus guernei

Régimbart, 1891

http://azoresbioportal.uac.pt/azores-species/hydroporus-guernei-7411/

##### Ecological interactions

###### Native status

Azores endemic

##### Distribution

COR; FLO; FAI; PIC; SJG; TER; SMG; SMR

##### Notes

Biogeographical Realm: Western Palearctic (Macaronesia)

#### Elateridae

#### Aeolus melliculus moreleti

Tarnier, 1860

http://azoresbioportal.uac.pt/azores-species/aeolus-melliculus-moreleti-13463/

##### Ecological interactions

###### Native status

Introduced

##### Distribution

FLO; FAI; GRA; SJG; TER; SMG; SMR

##### Notes

Biogeographical Realm: Neotropical

#### Alestrus dolosus

(Crotch, 1867)

http://azoresbioportal.uac.pt/azores-species/alestrus-dolosus-7551/

##### Ecological interactions

###### Native status

Azores endemic

##### Distribution

FLO; FAI*; PIC*; TER; SMG; SMR

##### Notes

Biogeographical Realm: Western Palearctic (Macaronesia)

#### Athous pomboi

Platia & Borges, 2002

http://azoresbioportal.uac.pt/azores-species/athous-pomboi-6826/

##### Ecological interactions

###### Native status

Azores endemic

##### Distribution

SMR

##### Notes

Biogeographical Realm: Western Palearctic (Macaronesia)

#### Hydrophilidae

#### Cercyon haemorrhoidalis

(Fabricius, 1775)

http://azoresbioportal.uac.pt/azores-species/cercyon-haemorrhoidalis-7400/

##### Ecological interactions

###### Native status

Introduced

##### Distribution

FLO; FAI; PIC; GRA; SJG; TER; SMG; SMR

##### Notes

Biogeographical Realm: Holarctic

#### Lathridiidae

#### Cartodere bifasciata

Reitter, 1877

http://azoresbioportal.uac.pt/azores-species/cartodere-bifasciata-7673/

##### Ecological interactions

###### Native status

Introduced

##### Distribution

FAI; GRA; TER*; SMG; SMR

##### Notes

Also present: MAD (Biogeographical Realm: Cosmopolitan)

#### Cartodere nodifer

(Westwood, 1839)

http://azoresbioportal.uac.pt/azores-species/cartodere-nodifer-7165/

##### Ecological interactions

###### Native status

Introduced

##### Distribution

FLO; FAI; PIC; GRA; SJG*; TER; SMG; SMR

##### Notes

Also present: MAD; CAN (Biogeographical Realm: Cosmopolitan)

#### Cartodere satelles

(Blackburn, 1888)

http://azoresbioportal.uac.pt/azores-species/cartodere-satelles-9639/

##### Ecological interactions

###### Native status

Introduced

##### Distribution

TER*; SMR*

##### Notes

Also present: MAD (Biogeographical Realm: Cosmopolitan)

#### Metophthalmus occidentalis

Israelson, 1984

http://azoresbioportal.uac.pt/azores-species/metophthalmus-occidentalis-7663/

##### Ecological interactions

###### Native status

Azores endemic

##### Distribution

FAI; GRA; SMG; SMR

##### Notes

Biogeographical Realm: Cosmopolitan

#### Leiodidae

#### Catops coracinus

Kellner, 1846

http://azoresbioportal.uac.pt/azores-species/catops-coracinus-7091/

##### Ecological interactions

###### Native status

Native

##### Distribution

FAI*; GRA; SJG*; TER*; SMG*

##### Notes

Biogeographical Realm: Cosmopolitan

#### Monotomidae

#### Rhizophagus ferrugineus

(Paykull, 1800)

http://azoresbioportal.uac.pt/azores-species/rhizophagus-ferrugineus-7068/

##### Ecological interactions

###### Native status

Introduced

##### Distribution

TER*

##### Notes

Also present: CAN (Biogeographical Realm: Cosmopolitan)

#### Mycetophagidae

#### Typhaea stercorea

(Linnaeus, 1758)

http://azoresbioportal.uac.pt/azores-species/typhaea-stercorea-7676/

##### Ecological interactions

###### Native status

Introduced

##### Distribution

FLO; FAI; PIC; GRA; TER; SMG; SMR

##### Notes

Also present: MAD; CAN; CVP (Biogeographical Realm: Cosmopolitan)

#### Nitidulidae

#### Carpophilus fumatus

(Boheman, 1851)

http://azoresbioportal.uac.pt/azores-species/carpophilus-fumatus-7562/

##### Ecological interactions

###### Native status

Introduced

##### Distribution

COR; FLO; FAI; PIC; GRA; SJG; TER; SMG; SMR

##### Notes

Also present: MAD; CVP (Biogeographical Realm: Cosmopolitan)

#### Carpophilus hemipterus

(Linnaeus, 1758)

http://azoresbioportal.uac.pt/azores-species/carpophilus-hemipterus-7580/

##### Ecological interactions

###### Native status

Introduced

##### Distribution

FAI; GRA; SJG; TER; SMG; SMR

##### Notes

Also present: MAD; CAN; CVP (Biogeographical Realm: Cosmopolitan)

#### Epuraea biguttata

(Thunberg, 1784)

http://azoresbioportal.uac.pt/azores-species/epuraea-biguttata-7616/

##### Ecological interactions

###### Native status

Introduced

##### Distribution

FLO*; FAI; PIC; GRA; SJG; TER; SMG; SMR

##### Notes

Also present: MAD; CAN (Biogeographical Realm: Palearctic)

#### Meligethes aeneus

(Fabricius, 1775)

http://azoresbioportal.uac.pt/azores-species/meligethes-aeneus-7606/

##### Ecological interactions

###### Native status

Introduced

##### Distribution

FLO; FAI; PIC; SJG; TER; SMG; SMR

##### Notes

Also present: MAD; CAN (Biogeographical Realm: Holarctic)

#### Stelidota geminata

(Say, 1825)

http://azoresbioportal.uac.pt/azores-species/stelidota-geminata-7618/

##### Ecological interactions

###### Native status

Introduced

##### Distribution

FLO; FAI*; PIC*; GRA; SJG*; TER*; SMR*

##### Notes

Biogeographical Realm: Neotropical

#### Phalacridae

#### Stilbus testaceus

(Panzer, 1797)

http://azoresbioportal.uac.pt/azores-species/stilbus-testaceus-7480/

##### Ecological interactions

###### Native status

Native

##### Distribution

FLO; FAI; GRA; SJG; TER; SMG; SMR

##### Notes

Also present: MAD; CAN (Biogeographical Realm: Western Palearctic)

#### Ptiliidae

#### Ptenidium pusillum

(Gyllenhal, 1808)

http://azoresbioportal.uac.pt/azores-species/ptenidium-pusillum-7320/

##### Ecological interactions

###### Native status

Introduced

##### Distribution

FLO; FAI; PIC; TER; SMG; SMR

##### Notes

Also present: MAD; CAN (Biogeographical Realm: Cosmopolitan)

#### Scarabaeidae

#### Onthophagus taurus

(Schreber, 1759)

http://azoresbioportal.uac.pt/azores-species/onthophagus-taurus-7532/

##### Ecological interactions

###### Native status

Introduced

##### Distribution

COR; FLO; FAI; PIC; GRA; SJG; TER; SMG; SMR

##### Notes

Biogeographical Realm: Palearctic

#### Scraptiidae

#### Anaspis proteus

Wollaston, 1854

http://azoresbioportal.uac.pt/azores-species/anaspis-proteus-7855/

##### Ecological interactions

###### Native status

Native

##### Distribution

COR; FLO; FAI; PIC; GRA; SJG; TER; SMG; SMR

##### Notes

Also present: MAD; CAN (Biogeographical Realm: Western Palearctic (Macaronesia))

#### Silvanidae

#### Cryptamorpha desjardinsii

(Guérin-Méneville, 1844)

http://azoresbioportal.uac.pt/azores-species/cryptamorpha-desjardinsii-7620/

##### Ecological interactions

###### Native status

Introduced

##### Distribution

COR; FLO; FAI; PIC; GRA; SJG; TER; SMG; SMR

##### Notes

Also present: MAD; CAN (Biogeographical Realm: Western Palearctic)

#### Trichoptera

#### Limnephilidae

#### Limnephilus atlanticus

Nybom, 1948

http://azoresbioportal.uac.pt/azores-species/limnephilus-atlanticus-8582/

##### Ecological interactions

###### Native status

Azores endemic

##### Distribution

COR; FLO*; FAI; PIC*; SJG; TER*; SMG*

##### Notes

Biogeographical Realm: Western Palearctic (Macaronesia)

#### Lepidoptera

#### Crambidae

#### Eudonia luteusalis

(Hampson, 1907)

http://azoresbioportal.uac.pt/azores-species/eudonia-luteusalis-8816/

##### Ecological interactions

###### Native status

Azores endemic

##### Distribution

FLO; FAI; PIC; SJG; TER; SMG; SMR

##### Notes

Biogeographical Realm: Western Palearctic (Macaronesia)

#### Scoparia coecimaculalis

Warren, 1905

http://azoresbioportal.uac.pt/azores-species/scoparia-coecimaculalis-8821/

##### Ecological interactions

###### Native status

Azores endemic

##### Distribution

FLO*; FAI; PIC; GRA; SJG; TER; SMG; SMR

##### Notes

Biogeographical Realm: Western Palearctic (Macaronesia)

#### Scoparia semiamplalis

Warren, 1905

http://azoresbioportal.uac.pt/azores-species/scoparia-semiamplalis-8822/

##### Ecological interactions

###### Native status

Azores endemic

##### Distribution

FLO; FAI; PIC; SJG; TER; SMG; SMR

##### Notes

Biogeographical Realm: Western Palearctic (Macaronesia)

#### Gelechiidae

#### Brachmia infuscatella

Rebel, 1940

http://azoresbioportal.uac.pt/azores-species/brachmia-infuscatella-8738/

##### Ecological interactions

###### Native status

Azores endemic

##### Distribution

FAI; PIC; SJG; TER; SMR*

##### Notes

Biogeographical Realm: Western Palearctic (Macaronesia)

#### Geometridae

#### Ascotis fortunata azorica

Pinker, 1971

http://azoresbioportal.uac.pt/azores-species/ascotis-fortunata-azorica-13542/

##### Ecological interactions

###### Native status

Azores endemic

##### Distribution

COR; FLO; FAI; PIC; GRA; SJG; TER; SMG; SMR

##### Notes

Biogeographical Realm: Western Palearctic (Macaronesia)

#### Cyclophora azorensis

(Prout, 1920)

http://azoresbioportal.uac.pt/azores-species/cyclophora-azorensis-8745/

##### Ecological interactions

###### Native status

Azores endemic

##### Distribution

COR; FLO; FAI; PIC; GRA; SJG; TER; SMG; SMR

##### Notes

Biogeographical Realm: Western Palearctic (Macaronesia)

#### Cyclophora puppillaria granti

(Prout, 1935)

http://azoresbioportal.uac.pt/azores-species/cyclophora-puppillaria-granti-13543/

##### Ecological interactions

###### Native status

Azores endemic

##### Distribution

SMR

##### Notes

Biogeographical Realm: Western Palearctic (Macaronesia)

#### Nycterosea obstipata

(Fabricius, 1794)

http://azoresbioportal.uac.pt/azores-species/nycterosea-obstipata-8749/

##### Ecological interactions

###### Native status

Native

##### Distribution

FLO; FAI; PIC; GRA; SJG; TER; SMG; SMR

##### Notes

Also present: MAD; CAN; CVP (Biogeographical Realm: Cosmopolitan)

#### Xanthorhoe inaequata

Warren, 1905

http://azoresbioportal.uac.pt/azores-species/xanthorhoe-inaequata-8750/

##### Ecological interactions

###### Native status

Azores endemic

##### Distribution

COR; FLO; FAI; PIC; GRA; SJG; TER; SMG; SMR

##### Notes

Biogeographical Realm: Western Palearctic (Macaronesia)

#### Gracillariidae

#### Caloptilia schinella

(Walsingham, 1908)

http://azoresbioportal.uac.pt/azores-species/caloptilia-schinella-8753/

##### Ecological interactions

###### Native status

Introduced

##### Distribution

COR; FAI; PIC; SJG; TER; SMG; SMR

##### Notes

Also present: MAD; CAN (Biogeographical Realm: Cosmopolitan)

#### Micrurapteryx bistrigella

(Rebel, 1940)

http://azoresbioportal.uac.pt/azores-species/micrurapteryx-bistrigella-8754/

##### Ecological interactions

###### Native status

Azores endemic

##### Distribution

FLO; PIC; SJG; TER*

##### Notes

Biogeographical Realm: Western Palearctic (Macaronesia)

#### Phyllocnistis citrella

Stainton, 1856

http://azoresbioportal.uac.pt/azores-species/phyllocnistis-citrella-8759/

##### Ecological interactions

###### Native status

Introduced

##### Distribution

FLO; FAI; PIC; GRA; SJG; TER; SMG; SMR

##### Notes

Also present: MAD; CAN (Biogeographical Realm: Cosmopolitan)

#### Noctuidae

#### Agrotis ipsilon

(Hufnagel, 1766)

http://azoresbioportal.uac.pt/azores-species/agrotis-ipsilon-8763/

##### Ecological interactions

###### Native status

Native

##### Distribution

COR; FLO; FAI; PIC; GRA; SJG; TER; SMG; SMR

##### Notes

Also present: MAD; CAN; CVP (Biogeographical Realm: Cosmopolitan)

#### Autographa gamma

(Linnaeus, 1758)

http://azoresbioportal.uac.pt/azores-species/autographa-gamma-8765/

##### Ecological interactions

###### Native status

Native

##### Distribution

COR; FLO; FAI; PIC; GRA; SJG; TER; SMG; SMR

##### Notes

Also present: MAD; CAN (Biogeographical Realm: Palearctic)

#### Chrysodeixis chalcites

(Esper, 1789)

http://azoresbioportal.uac.pt/azores-species/chrysodeixis-chalcites-8766/

##### Ecological interactions

###### Native status

Native

##### Distribution

COR; FLO; FAI; PIC; GRA; SJG; TER; SMG; SMR

##### Notes

Also present: MAD; CAN; CVP (Biogeographical Realm: Western Palearctic)

#### Mesapamea storai

(Rebel, 1940)

http://azoresbioportal.uac.pt/azores-species/mesapamea-storai-8778/

##### Ecological interactions

###### Native status

Azores endemic

##### Distribution

COR; FLO; FAI; PIC; GRA; SJG; TER; SMG

##### Notes

Biogeographical Realm: Western Palearctic (Macaronesia)

#### Mythimna unipuncta

(Haworth, 1809)

http://azoresbioportal.uac.pt/azores-species/mythimna-unipuncta-8780/

##### Ecological interactions

###### Native status

Native

##### Distribution

COR; FLO; FAI; PIC; GRA; SJG; TER; SMG; SMR

##### Notes

Also present: MAD; CAN (Biogeographical Realm: Cosmopolitan)

#### Phlogophora interrupta

(Warren, 1905)

http://azoresbioportal.uac.pt/azores-species/phlogophora-interrupta-8761/

##### Ecological interactions

###### Native status

Azores endemic

##### Distribution

FLO; FAI; PIC; GRA; SJG; TER; SMG; SMR

##### Notes

Biogeographical Realm: Western Palearctic (Macaronesia)

#### Xestia c-nigrum

(Linnaeus, 1758)

http://azoresbioportal.uac.pt/azores-species/xestia-c-nigrum-8792/

##### Ecological interactions

###### Native status

Native

##### Distribution

COR; FLO; FAI; PIC; GRA; SJG; TER; SMG; SMR

##### Notes

Also present: MAD (Biogeographical Realm: Holarctic)

#### Nymphalidae

#### Hipparchia azorina occidentalis

(Sousa, 1985)

http://azoresbioportal.uac.pt/azores-species/hipparchia-azorina-occidentalis-13545/

##### Ecological interactions

###### Native status

Azores endemic

##### Distribution

COR; FLO

##### Notes

Biogeographical Realm: Western Palearctic (Macaronesia)

#### Hipparchia miguelensis

(Le Cerf, 1935)

http://azoresbioportal.uac.pt/azores-species/hipparchia-miguelensis-8798/

##### Ecological interactions

###### Native status

Azores endemic

##### Distribution

SMG

##### Notes

Biogeographical Realm: Western Palearctic (Macaronesia)

#### Tineidae

#### Oinophila v-flava

(Haworth, 1828)

http://azoresbioportal.uac.pt/azores-species/oinophila-v-flava-8833/

##### Ecological interactions

###### Native status

Introduced

##### Distribution

FLO; FAI; PIC*; TER; SMG

##### Notes

Also present: MAD; CAN (Biogeographical Realm: Palearctic)

#### Opogona sacchari

(Bojer, 1856)

http://azoresbioportal.uac.pt/azores-species/opogona-sacchari-8838/

##### Ecological interactions

###### Native status

Introduced

##### Distribution

COR; FAI; PIC; GRA; SJG*; TER; SMG; SMR

##### Notes

Also present: MAD; CAN; CVP (Biogeographical Realm: Cosmopolitan)

#### Tortricidae

#### Rhopobota naevana

(Hübner, 1817)

http://azoresbioportal.uac.pt/azores-species/rhopobota-naevana-8858/

##### Ecological interactions

###### Native status

Introduced

##### Distribution

FLO*; FAI*; PIC; GRA; SJG*; TER*; SMG*; SMR*

##### Notes

Biogeographical Realm: Holarctic

#### Yponomeutidae

#### Argyresthia atlanticella

Rebel, 1940

http://azoresbioportal.uac.pt/azores-species/argyresthia-atlanticella-8859/

##### Ecological interactions

###### Native status

Azores endemic

##### Distribution

COR; FLO; FAI; PIC; GRA; SJG; TER; SMG; SMR

##### Notes

Biogeographical Realm: Western Palearctic (Macaronesia)

## Analysis

**Azorean Arthropod biodiversity - towards a more complete knowledge**

**Figure 2.**
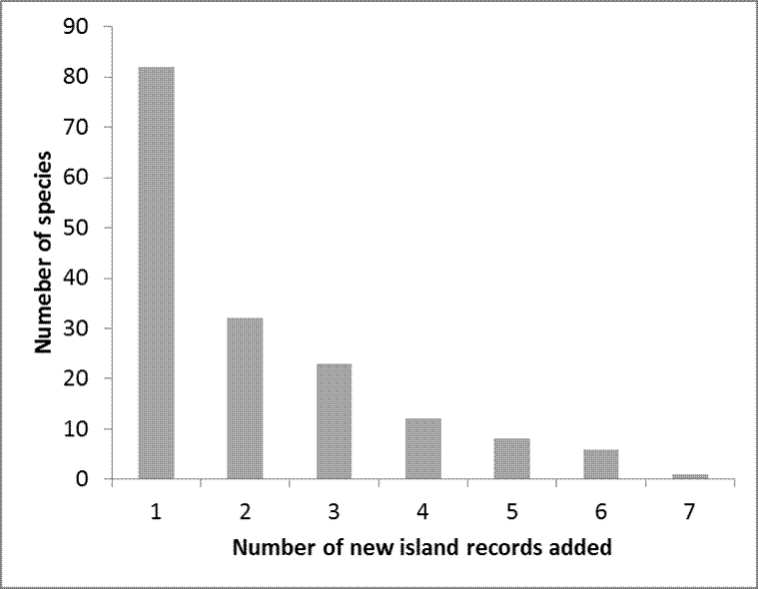
Frequency distribution of the number of new island records per species.

The ultimate goal of biodiversity assessments is documenting all species inhabiting a region. However, this has often proven impossible to achieve given the unfeasibility of collecting every single species that exists in a study area. This study focuses on the terrestrial arthropod diversity of the Azores and encompasses most orders of the phylum Arthropoda. A pool of a total of 1215 species and subspecies was surveyed, representing 53% of the whole arthropod fauna known from the Azores (Borges et al. 2010). By deliberately not surveying Crustacea, Acari, Collembola, Diptera and Hymenoptera, we excluded 47% of the archipelago's species pool. Yet, this study added 10 endemic and at least 16 other species, mostly exotics, to the known Azorean arthropod fauna. More will be added soon after the on-going revision of Staphylinidae (in prep.) and Zopheridae (Borges et al. 2016, in press). Overall, at least 26 species that occur in native forests were added to the Azorean arthropod fauna list. The new 346 taxonomic records provided by this study (see Suppl. material 4 for the complete list of new records per island) represent on average an increase in species number of about 10% for each studied island (Table 2). However, the increment for São Jorge island was about 22%, while for São Miguel this represented only 3% (Table 2). 164 species were found in new islands, with an average of two islands per species. For 82 of those species only one new island was added to their known distribution contrasting with 27 species for which four or more islands were added (Fig. 2). Notably, nine out of the 27 species with more than three island added to their previous distribution belong to Arachnida. In fact, arachnids but also millipedes and centipedes experienced a large proportion of new records (more than 30%) (see Table 3).

**Figure 3.**
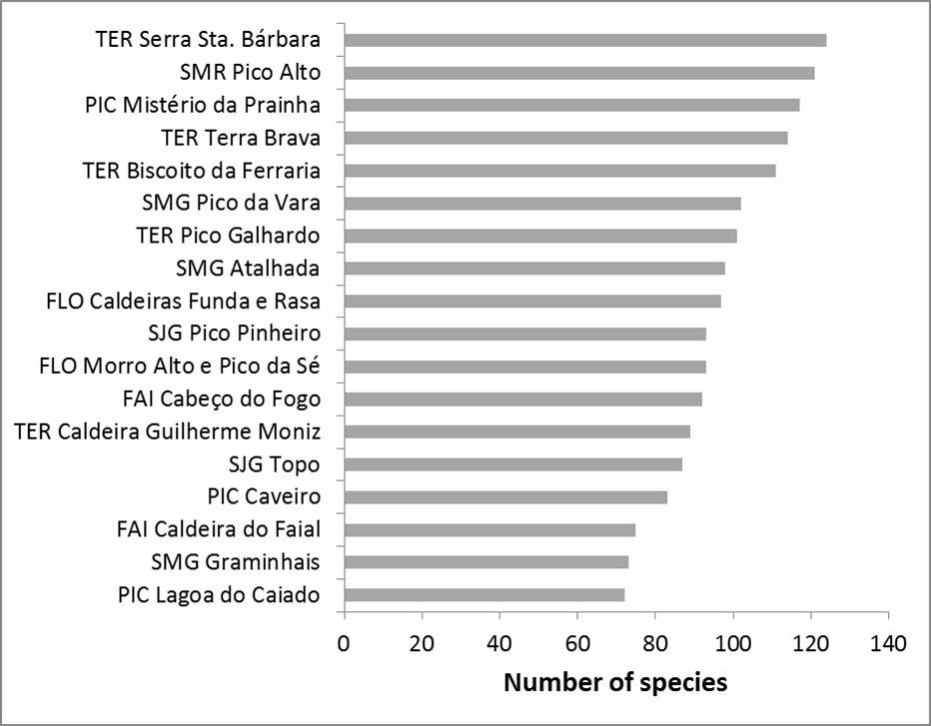
Number of species per native forest fragments. Island codes as in Table 1

The number of species identified for each of the 18 native forest fragments surveyed is shown in Fig. 3. The fragment with the highest species diversity is Serra de Santa Bárbara in Terceira island (S = 124), which is also the larger native forest area in the Azores. Remarkably, one of the smallest fragments, Pico Alto in Santa Maria island, is the second most diverse (S = 121).

**Figure 4.**
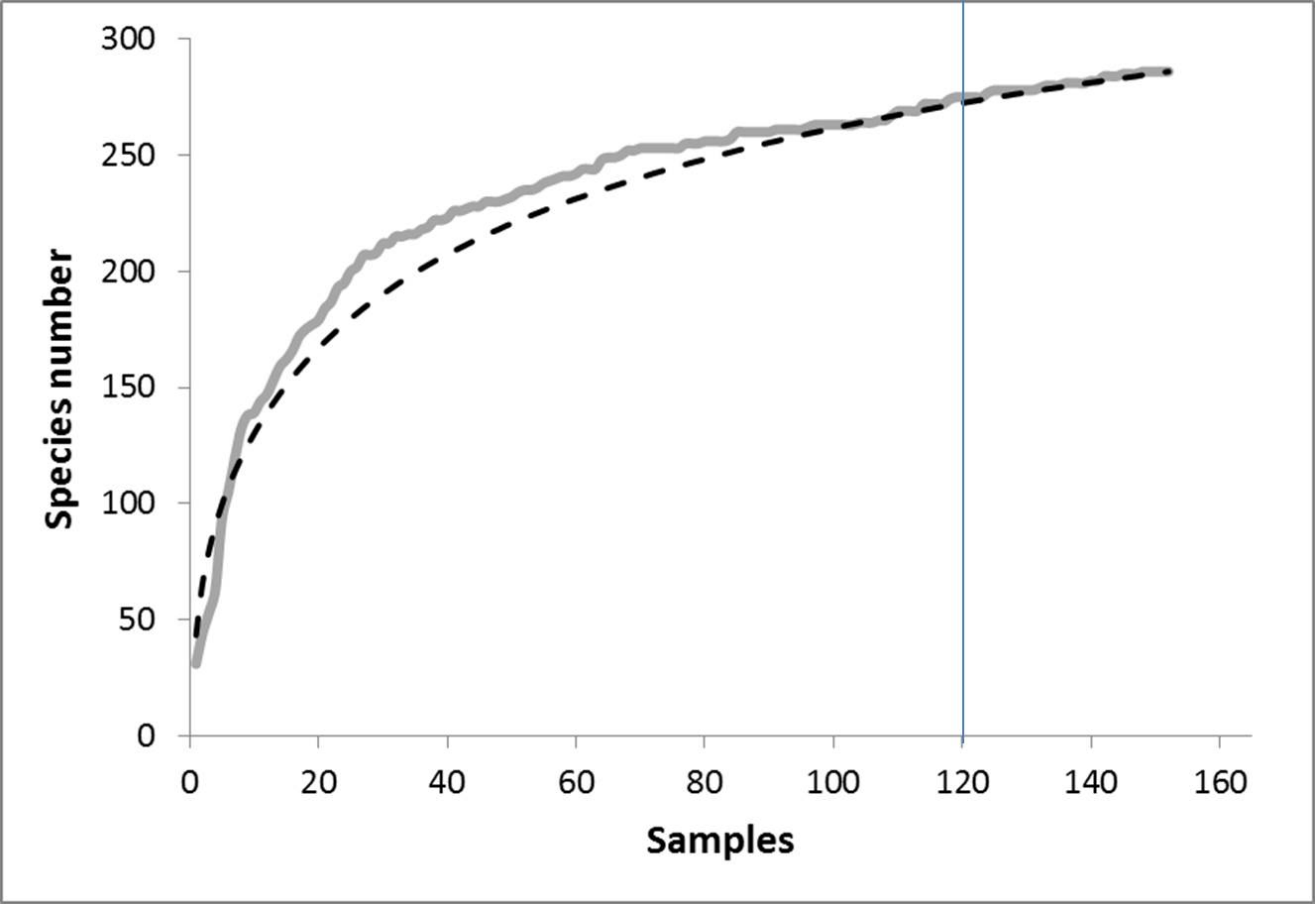
Species accumulation curve for the 286 species of arthropods collected in 152 pitfall and beating samples between 1999 and 2011. The solid line corresponds to the chronological sample sequence and the dotted line is a randomized curve (1000 runs). Samples to the left of the vertical line were collected in BALA1 and to the right in BALA2.

BALA2 samples only added 4% of species to the previous BALA survey (Fig. 4). Interestingly, 59 samples collected in the first two years of survey (1999 and 2000) provided about 81% of the total species recorded in this study.

### The most abundant species

**Figure 5.**
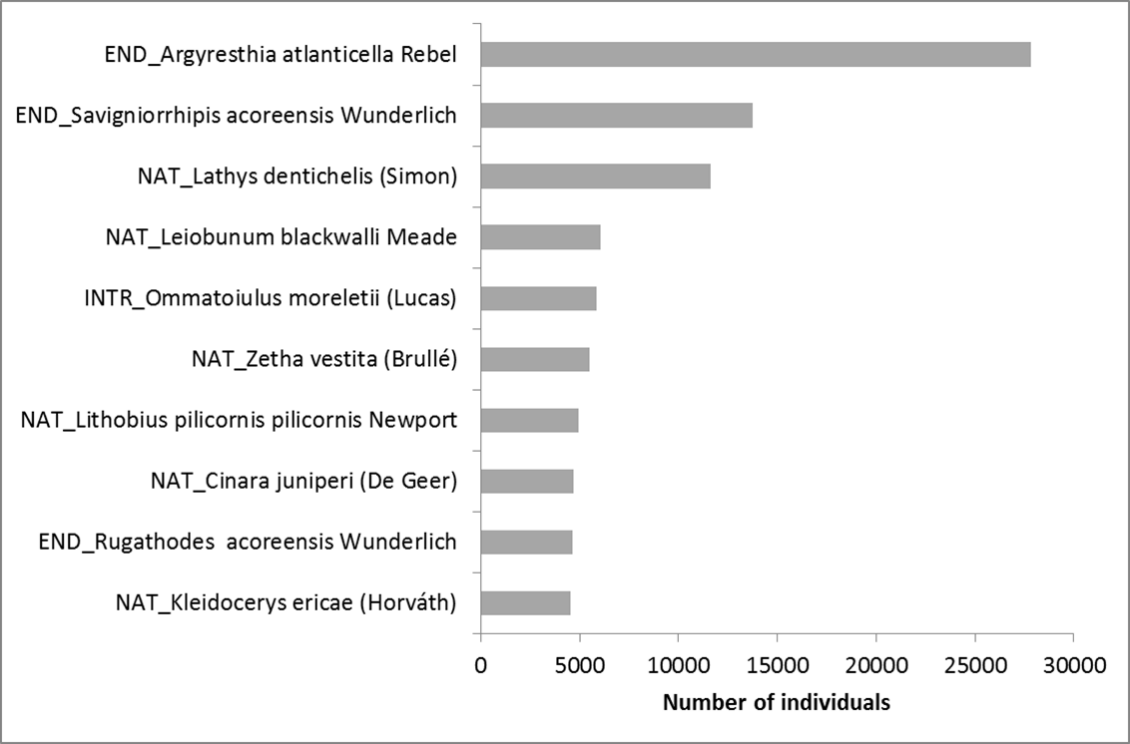
The ten most abundant species in the database. END - endemic from Azores; NAT - native non-endemic species; INTR - species introduced in the archipelago.

**Figure 6.**
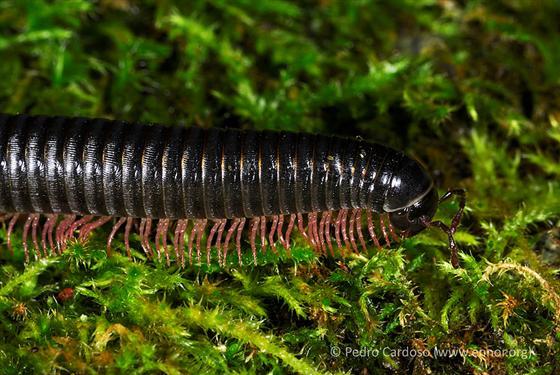
The millipede *Ommatoiulus moreletii* (Credit: Pedro Cardoso)

**Figure 7.**
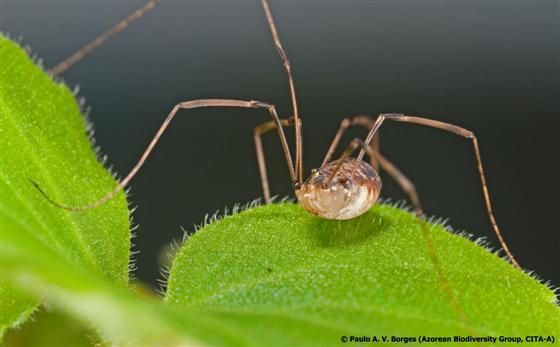
The opilion *Leiobunum blackwalli* (Credit: Paulo A.V. Borges).

**Figure 8.**
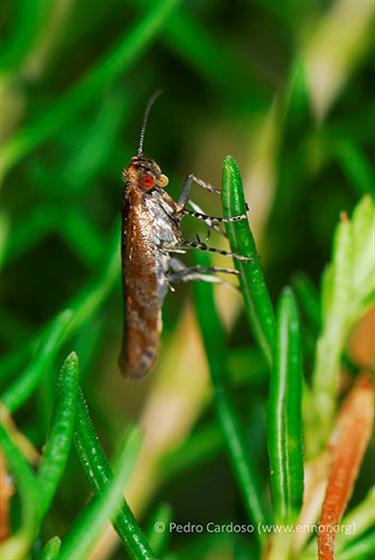
The moth *Argyresthia atlanticella* (Credit: Paulo A.V. Borges)

**Figure 9.**
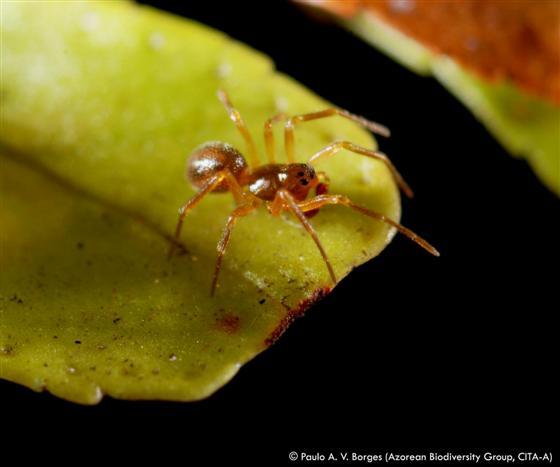
The spider *Savigniorrhipis acoreensis* (Credit: Paulo A.V. Borges)

A total of 163744 individuals were identified as belonging to the 286 species (see Suppl. material 5 for the complete list of abundance per species). The ten most abundant species (Fig. 5) accommodate 56% of the total number of individuals and include mostly indigenous species (endemic or native non-endemic). The single introduced species is the millipede *Ommatoiulus moreletii* (*Fig. 6*). With exception of the millipede *Ommatoiulus moreletii*, the centipede *Lithobius pilicornis pilicornis* and the opilion *Leiobunum blackwalli* (Fig. 7) that are mostly soil epigean species, the other seven species live preferentially in the canopies of Azorean endemic trees. The moth *Argyresthia atlanticella* (Fig. 8) is particularly common in *Juniperus brevifolia* and *Erica azorica*; the spider *Savigniorrhipis acoreensis* (Fig. 9) is particularly abundant in *Juniperus brevifolia*, but can also be found in other plants

## Discussion

Cardoso et al. (2011) identified seven impediments in invertebrate conservation. Three of them are particularly relevant for our study: most species are undescribed (the Linnean shortfall), the distribution of described species is mostly unknown (the Wallacean shortfall), and the abundance of species and its variation in space and time are unknown (the Prestonian shortfall). We argue that with the BALA project we were able to contribute to overcome some of these impediments in the Azores. In fact, we show that as a result of the standardized sampling performed in Azorean native forests we were able to: i) decrease the Linnean shortfall, by increasing the number of described Azorean endemics (e.g. Blas and Borges 1999, Ribes and Borges 2001, Platia and Borges 2002, Quartau and Borges 2003, Borges et al. 2004, Borges and Wunderlich 2008, Crespo et al. 2013, Crespo et al. 2014, Borges et al. 2016); ii) decrease the Wallacean shortfall, by increasing the known distribution of many endemic and exotic species in the archipelago (e.g. Borges et al. 2005a, Borges et al. 2006, Cardoso et al. 2009, Meijer et al. 2011); and iii) decrease the Prestonian shortfall, by using standardized sampling, which allowed the comparison of species abundances in space and time as many of the same sites were sampled in two different time periods.

The increase in the number of islands from where each species is known and the distribution increase for many species within each island shows the importance of regional standardized surveys, which provided a major improvement in the knowledge of the distribution of arthropod species in the native forests of the Azores.

The fact that most diversity was captured during the first two years of the project reflects the importance of sampling a wide geographic range covering all the islands and the maximum number of sites. Increasing the number of samples per fragment (sampling performed in 2004) or replicating the sampling at a different time (29 sites in 2010 to 2011; BALA2 project) had a lesser impact in increasing our knowledge about biodiversity (Fig. 4).

The future agenda for surveying and monitoring Azorean arthropod biodiversity includes:

a) expanding the standardized survey of Azorean arthropods to other habitat types, mostly man-modified, an already on-going task for some of the islands (see e.g. Cardoso et al. 2009, Meijer et al. 2011, Cardoso et al. 2013, Florencio et al. 2013, Santos et al. 2010);

b) selecting study areas along a comprehensive environmental gradient where an optimal sampling strategy will be applied in order to sample the entire arthropod communities (All Taxa Biodiversity Inventory - ATBI). ATBIs are intensive sampling efforts to identify and record all living species that exist within a given area and simultaneously create a common and standardized biodiversity database (Lawton and Gaston 2001);

c) finishing the identification of many morphospecies. Good progress has been made with Staphylinidade (Borges et al. in prep.), but other taxa need further effort to reach proper identification;

d) increase sampling and update the current list of Azorean Hymenoptera and Diptera, which is clearly incomplete (Borges et al. 2010). The shortage of taxonomists who can adequately identify species (i.e. the so-called *Taxonomic Impediment*) has prevented advances in the knowledge for many diverse groups in the Azores, including these two.

e) contributing to the validation and updating of the pan-European checklists programs, including Fauna Europaea (Jong et al. 2014) and PESI (Jong et al. 2015) allowing a more general evaluation and comparison of species distributions and statuses.

This study advances the knowledge on the unique arthropod biodiversity of the Azores, but at the same time highlights the need for further surveys. We strongly believe that the BALA project will stimulate further research and conservation actions towards the preservation of Azorean biodiversity. Furthermore, we hope that all the taxa yet to be identified will entice taxonomist to join us in the endeavour of cataloguing all terrestrial arthropods of the most remote of the Macaronesian archipelagos, the Azores. The ongoing longterm research projects in Azores and the recent creation of the E-Repository ISLANDLAB will create new opportunities for biodiversity studies in Azores.

## Supplementary Material

Supplementary material 1 Appendix 1 - Detailed data on the distribution and abundance of the studied species Data type: Occurrences and abundance
Brief description: Detailed data on the occurrences and abundances of the studied species. Data on species abundance in each individual sample (pitfall trap or canopy beating) for the 152 transects in eighteen protected areas and seven Azorean islands.
File: oo_114229.xlsx Borges et al.

Supplementary material 2 Appendix 2 - Metadata from Appendix 1 Data type: Text in pdf
Brief description: METADATA from Appendix 1 – Detailed data on the distribution and abundance of the studied species
File: oo_114225.pdf Borges et al.

Supplementary material 3 Appendix 3 - Sites UTM coordinates Data type: Sites coordinates
Brief description: UTM coordinates (regions 25S for Flores and 26S for all other islands), altitude (meters) and supporting project of the studied transects in the Azores. Transect code according to island, reserve and transect number (see text)
File: oo_114224.xlsx Borges et al.

Supplementary material 4 Appendix 4. Complete list of new records per island. Data type: Occurrences
Brief description: The complete list of new records per island.
File: oo_114227.xlsx Borges et al.

Supplementary material 5 Appendix 5 -Abundance data Data type: Abundance data
Brief description: Detailed abundance for each species in each of the 18 protected areas
File: oo_114228.xlsx Borges et al.

## Figures and Tables

**Table 1.** Main characteristics of the Azorean islands (bold) and native forest fragments sampled from 1999 to 2011, including area (hectares), highest point (altitude in metres), distance to the nearest island/fragment (isolation in kilometres) and the oldest geological age of emerged substrate (million years BP) (adapted from Gaspar et al. 2008).

Island	Fragment	Code	Area (ha)	Altitude (m)	Isolation (km)	Age (my)
**Flores**		**FLO**	**14102**	**911**	**236.43**	**2.16**
	Morro Alto e Pico da Sé	MO	1331	911	6.02	2.16
	Caldeiras Funda e Rasa	FR	240	773	6.02	2.16
**Faial**		**FAI**	**17306**	**1043**	**34.26**	**0.73**
	Caldeira do Faial	CA	190	934	4.67	0.73
	Cabeço do Fogo	CG	36	597	4.67	0.60
**Pico**		**PIC**	**44498**	**2350**	**32.42**	**0.30**
	Mistério da Prainha	MP	689	881	2.92	0.26
	Caveiro	CA	184	1077	4.61	0.27
	Lagoa do Caiado	LC	79	945	2.92	0.28
**São Jorge**		**SJG**	**24365**	**1053**	**32.42**	**0.55**
	Topo	TO	220	946	15.13	0.55
	Pico Pinheiro	PP	73	717	15.13	0.55
**Terceira**		**TER**	**40030**	**1021**	**71.67**	**3.52**
	S. Bárbara e M. Negros	SB	1347	1021	7.20	1.24
	Biscoito da Ferraria	BF	557	809	3.03	0.10
	Guilherme Moniz	GM	223	487	2.70	0.41
	Terra Brava	TB	180	726	2.70	0.10
	Pico do Galhardo	PG	38	655	2.79	0.10
**São Miguel**		**SMG**	**74456**	**1105**	**97.53**	**4.01**
	Pico da Vara	PV	306	1105	3.42	3.20
	Graminhais	GR	15	930	4.02	3.20
	Atalhada	AT	10	500	3.42	4.01

**Table 2.** Species richness for the Azores archipelago and each island. Total currently known species, the number of species surveyed during this study and those that represent new records are presented.

	Known species in the Azores	Pool of surveyed taxa	New records	New records (%)
AZORES	2316	1215	26	2.13
FLO	797	461	55	11.93
FAI	945	537	51	9.49
PIC	808	463	46	9.93
SJG	620	359	76	21.17
TER	1224	731	52	7.11
SMG	1592	861	28	3.25
SMR	799	573	38	6.63

**Table 3.** Total species and subspecies records for the Azores, new species and subspecies records during this study and increment for the most speciose classes and orders. Values for all islands are added, so richness may be up to 7 times higher than the archipelago's richness (as 7 islands were surveyed). (*)The Coleoptera families Staphylinidae and Zopheridae were not considered (see text).

	Total records	New records	New Records (%)
**Class Arachnida**	**362**	**124**	**34.25**
Order Pseudoscorpiones	19	5	26.32
Order Opiliones	12	11	91.67
Order Araneae	331	108	32.63
**Class Diplopoda**	**67**	**24**	**35.82**
Order Polydesmida	18	8	44.44
Order Polyxenida	0	0	0.00
Order Julida	44	12	27.77
Order Chordeumatida	5	4	80.00
**Class Chilopoda**	**21**	**9**	**42.86**
Order Scutigeromorpha	0	0	0.00
Order Lithobiomorpha	7	0	0.00
Order Scolopendromorpha	4	2	50.00
Order Geophilomorpha	10	7	70.00
**Class Insecta**	**1012**	**189**	**18,68**
Order Microcoryphia	13	4	30.77
Order Zygentoma	0	0	0.00
Order Ephemeroptera	6	0	0.00
Order Odonata	0	0	0.00
Order Blattaria	7	3	42.86
Order Orthoptera	10	0	0.00
Order Phasmatodea	0	0	0.00
Order Dermaptera	14	0	0.00
Order Psocoptera	75	40	53.33
Order Thysanoptera	76	6	7.89
Order Hemiptera	290	82	28.28
Order Neuroptera	7	3	42.86
Order Coleoptera (*)	361	36	9.97
Order Trichoptera	6	4	66.67
Order Lepidoptera	147	11	7.84
