# Supplementary material for: New records and detailed distribution and abundance of selected arthropod species collected between 1999 and 2011 in Azorean native forests
Source: Biodivers Data J. 2016 Dec 22;(4):e10948. doi: 10.3897/BDJ.4.e10948 (PMC5267528; doi:10.3897/BDJ.4.e10948)
Supplement: Supplementary material 2 — Appendix 2 - Metadata from Appendix 1 [file biodiversity_data_journal-4-e10948-s002.pdf]

## Supplementary Material 2

METADATA from Appendix 1 – Detailed data on the distribution and abundance of the studied species

**Column A- CODE**

All specimens were assigned a SITE CODE composed of several letters and numbers that read as follows

i) the first three letters refer to island (FLO – Flores; FAI – Faial; PIC – Pico; SJG – São Jorge; GRA – Graciosa; TER – Terceira; SMG – São Miguel; SMR – Santa Maria);

ii) the following two letters refer to fragment (Flores: FR - Caldeiras Funda e Rasa, MA - Morro Alto e Pico da Sé; Faial: CF – Caldeira do Faial, CG – Cabeço do Fogo; Pico: CA – Caveiro, LC – Lagoa do Caiado, MP – Mistério da Prainha; São Jorge: PP – Pico Pinheiro, TO – Topo; Terceira: BF – Biscoito da Ferraria, GM – Caldeira do Guilherme Moniz, PG – Pico do Galhardo, SB –Serra de Santa Bárbara, TB – Terra Brava; São Miguel: AT – Atalhada, GR – Graminhais, PV – Pico da Vara; Santa Maria: PA – Pico Alto);

iii) the following three characters refer to the sampling transect; and

iv) the next letter refers to the sampling technique: P - pitfall, B - canopy beating; for pitfall samples (P) TU – Turquin and ET – ethylene glycol; for canopy samples (B) the next two letters refer to the plant sampled: CA = *Calluna vulgaris*, CL = *Clethra arborea*, ER = *Erica azorica*, FR = *Frangula azorica*, IL = *Ilex perado* ssp. *azorica*, JU = *Juniperus brevifolia*, LA = *Laurus azorica*, MC = *Morella faya*, MS = *Myrsine africana*, PI = *Picconia azorica*, PT = *Pittosporum undulatum*, VA = *Vaccinium cylindraceum*.

v) The final numbers are the pitfall or beating sample.

**Column B - Fragment name**

**Column C - Site name (locality of the transect)**

**Column D - Site code**

i) the first three letters refer to island (FLO – Flores; FAI – Faial; PIC – Pico; SJG – São Jorge; GRA – Graciosa; TER – Terceira; SMG – São Miguel; SMR – Santa Maria);

ii) the following four letters refer to fragment (Flores: FR - Caldeiras Funda e Rasa, MA - Morro Alto e Pico da Sé; Faial: CF – Caldeira do Faial, CG – Cabeço do Fogo; Pico: CA – Caveiro, LC – Lagoa do Caiado, MP – Mistério da Prainha; São Jorge: PP – Pico Pinheiro, TO – Topo; Terceira: BF – Biscoito da Ferraria, GM – Caldeira do Guilherme Moniz, PG – Pico do Galhardo, SB –Serra de Santa Bárbara, TB – Terra Brava; São Miguel: AT – Atalhada, GR – Graminhais, PV – Pico da Vara; Santa Maria: PA – Pico Alto);

iii) the following three characters refer to the sampling transect;

**Column E – Azores Grid Zones**

**Column F – UTM E**

**Column G – UTM N**

**Column H – Project**

**Column I – Year**

**Column J – Month**

**Column K – Pitfall Solution /Plant**

**Column L – Sample code**

-The next letter refers to the sampling technique: P - pitfall, B - canopy beating;

-For pitfall samples (P) TU – Turquin and ET – ethylene glycol; for canopy samples (B) the next two letters refer to the plant sampled: CA = *Calluna vulgaris*, CL = *Clethra arborea*, ER = *Erica azorica*, FR = *Frangula azorica*, IL = *Ilex perado* ssp. *azorica*, JU = *Juniperus brevifolia*, LA = *Laurus azorica*, MC = *Morella faya*, MS = *Myrsine africana*, PI = *Picconia azorica*, PT = *Pittosporum undulatum*, VA = *Vaccinium cylindraceum*.

-The final numbers are the pitfall or beating sample.

**Column M – original morphospecies code pervious to species identification**

**Column N – Species name**

**Column O – Genus**

**Column P – restrictive specific**

**Column Q – subspecies**

**Column R – Authors**

**Column S – Family**

**Column T – Order**

**Column U – Class**

**Column V – Colonization Status**

E: Endemic from Azores
N: Native non-endemic
I: Introduced species

**Column W – Endemism Status**

MAC: endemic from Macaronesia
A_END: endemic from at least two islands
SIE- endemic from a single island

**Column X – Trophic Group**

F: fungivorous
H: herbivore
P: predator
S: saprophagous

**Column Y – AM/AF**

Number of adult specimens without sex identification

**Column Z – AM**

Number of male adult specimens

**Column AA – AF**

Number of female adult specimens

**Column AB – J**

Number of juvenile specimens

**Column AC – A/J**

Number of pre-adults (spiders) specimens

**Column AD – Total**

Total number of specimens

**Column AE – Adults**

Total number of adults
